# Predicting Progression from Mild Cognitive Impairment to Alzheimer's Dementia Using Clinical, MRI, and Plasma Biomarkers via Probabilistic Pattern Classification

**DOI:** 10.1371/journal.pone.0138866

**Published:** 2016-02-22

**Authors:** Igor O. Korolev, Laura L. Symonds, Andrea C. Bozoki, Alzheimer's Disease Neuroimaging Initiative

**Affiliations:** 1 Neuroscience Program, Michigan State University, East Lansing, Michigan, United States of America; 2 College of Osteopathic Medicine, Michigan State University, East Lansing, Michigan, United States of America; 3 Department of Neurology, Michigan State University, East Lansing, Michigan, United States of America; University of Manchester, UNITED KINGDOM

## Abstract

**Background:**

Individuals with mild cognitive impairment (MCI) have a substantially increased risk of developing dementia due to Alzheimer's disease (AD). In this study, we developed a multivariate prognostic model for predicting MCI-to-dementia progression at the individual patient level.

**Methods:**

Using baseline data from 259 MCI patients and a probabilistic, kernel-based pattern classification approach, we trained a classifier to distinguish between patients who progressed to AD-type dementia (n = 139) and those who did not (n = 120) during a three-year follow-up period. More than 750 variables across four data sources were considered as potential predictors of progression. These data sources included risk factors, cognitive and functional assessments, structural magnetic resonance imaging (MRI) data, and plasma proteomic data. Predictive utility was assessed using a rigorous cross-validation framework.

**Results:**

Cognitive and functional markers were most predictive of progression, while plasma proteomic markers had limited predictive utility. The best performing model incorporated a combination of cognitive/functional markers and morphometric MRI measures and predicted progression with 80% accuracy (83% sensitivity, 76% specificity, AUC = 0.87). Predictors of progression included scores on the Alzheimer's Disease Assessment Scale, Rey Auditory Verbal Learning Test, and Functional Activities Questionnaire, as well as volume/cortical thickness of three brain regions (left hippocampus, middle temporal gyrus, and inferior parietal cortex). Calibration analysis revealed that the model is capable of generating probabilistic predictions that reliably reflect the actual risk of progression. Finally, we found that the predictive accuracy of the model varied with patient demographic, genetic, and clinical characteristics and could be further improved by taking into account the confidence of the predictions.

**Conclusions:**

We developed an accurate prognostic model for predicting MCI-to-dementia progression over a three-year period. The model utilizes widely available, cost-effective, non-invasive markers and can be used to improve patient selection in clinical trials and identify high-risk MCI patients for early treatment.

## Introduction

Alzheimer’s disease (AD) is the leading cause of dementia in the aging population, affecting more than 30 million people worldwide [1]. AD is a degenerative brain disorder that causes a progressive decline in cognitive function, most notably memory loss, and other behavioral changes [2]. Individuals diagnosed with mild cognitive impairment (MCI) have a substantially increased risk of developing clinical AD, and MCI is often considered to be a transitional phase between healthy cognitive aging and dementia [3,4]. Thus, MCI represents a key prognostic and therapeutic target in the management of AD. However, MCI is a heterogeneous syndrome with varying clinical outcomes. Although up to 60% of MCI patients develop dementia within a ten-year period, many people remain cognitively stable or regain normal cognitive (NC) function [5,6].

Increasing efforts have focused on building predictive models of AD dementia using pattern classification methods based on clinical, imaging, genetic, and fluid biomarkers [7–11]. This line of research dates back to earlier studies from the late 1980s and 1990s, which tended to use more conventional statistical modeling methods or focus on univariate prediction, and were generally limited by relatively small sample sizes. For example, some earlier studies demonstrated the ability of baseline neuropsychological measures to predict dementia in cognitively impaired individuals [12–14]. Other earlier studies showed that baseline atrophy of the hippocampus or the surrounding medial temporal lobe regions, as measured using structural neuroimaging, could predict subsequent progression to dementia [15–17]. Prognostic classification of MCI at the individual patient level has the potential to improve clinical trial design, identify patients for early treatment, as well as guide clinical and patient decision-making. In this study, we develop a multivariate prognostic model [18] for predicting MCI-to-dementia progression using baseline data from the Alzheimer's Disease Neuroimaging Initiative (ADNI) [19]. We focus on using widely available, cost-effective, and minimally-invasive data sources, including: (a) clinical data, such as risk factors and cognitive / functional assessments; (b) morphometric measures derived from a structural magnetic resonance imaging (MRI) scan of the brain; and (c) blood plasma-based proteomic data. Much of this data is already routinely collected during the clinical workup of dementia and clinical trials.

We use a kernel-based classifier to predict future dementia status of MCI patients by incorporating heterogeneous (clinical, MRI, and proteomic) data. Kernel-based learning algorithms use “kernel functions” to encode the degree of similarity between examples in a dataset based on their features [20,21], such as individual MCI patients described by their unique biomarker patterns. We apply an extension of this methodology, known as multiple kernel learning (MKL), which allows integration of complementary information derived from different sources or representations of the data using separate kernels [22]. Recent studies suggest that multiple-kernel classifiers may integrate heterogeneous data more effectively than conventional single-kernel classifiers, improving classification of AD and MCI subjects by as much as 3–11% [23–25].

The prevailing approach in the literature has been to consider prediction of MCI-to-dementia progression as a non-probabilistic binary classification task, where all patients are unequivocally assigned to either the progressive MCI (P-MCI) or the non-progressive MCI (N-MCI) group [23,26–28]. Sir William Osler (1849–1919), a pre-eminent physician of the 20th century, is credited with stating that “medicine is a science of uncertainty and an art of probability” [29]. In this spirit, we adopt a recently proposed implementation of MKL that generates probabilistic predictions using Bayesian inference [30]. We anticipated that probabilistic prediction of MCI-to-dementia progression would provide clinically useful information beyond what is afforded by binary, non-probabilistic classification. Reliable probabilistic prediction would allow stratification of MCI patients into multiple groups according to the risk of progression. Alternatively, the probability associated with each individual prediction can be used as a measure of confidence, which in turn can be used to withhold the decision about future dementia status for ambiguous (“low confidence”) MCI cases. This approach is often referred to as classification with a “reject option” [31].

The objectives of this study were to determine whether: (a) clinical, MRI, and plasma proteomic data capture complementary information regarding the progression from MCI to dementia; (b) this information is more effectively learned using a multiple-kernel classifier as opposed to a single-kernel classifier; (c) the performance of our prognostic model is sensitive to patient heterogeneity; (d) model performance can be improved by taking into account the confidence of the predictions; and (e) the model's probabilistic predictions reflect any information regarding the time to progression for P-MCI patients.

## Materials and Methods

### Alzheimer’s Disease Neuroimaging Initiative (ADNI)

Data used in this study were obtained from the ADNI database (http://adni.loni.usc.edu). The ADNI was launched in 2003 by the National Institute on Aging (NIA), the National Institute of Biomedical Imaging and Bioengineering (NIBIB), the Food and Drug Administration (FDA), private pharmaceutical companies and non-profit organizations as a public-private partnership. ADNI is an observational study with both cross-sectional and longitudinal follow-up components. The primary goal of ADNI has been to test whether neuroimaging, fluid and genetic biomarkers, and cognitive assessments can be combined to measure the progression of MCI and early AD. The Principal Investigator of this initiative is Michael W. Weiner, MD, VA Medical Center and University of California–San Francisco. ADNI is the result of efforts of many co-investigators from a broad range of academic institutions and private corporations, and subjects have been recruited from over 50 sites across the U.S. and Canada. The first phase of ADNI (ADNI-1) was completed in 2010 and has been followed by ADNI-GO and ADNI-2. For up-to-date information, see www.adni-info.org.

In this study, we analyzed baseline visit data collected from MCI subjects who were recruited during ADNI-1. The various datasets were downloaded on or before the following dates: Clinical data–August 20, 2011; Structural MRI data–August 3, 2011; Plasma proteomic data–June 16, 2012. All subjects and their study partners completed the informed consent process, and the study protocols were reviewed and approved by the Institutional Review Board at each ADNI data collection site.

### Subjects

The general eligibility, inclusion, and exclusion criteria for ADNI subjects can be found on the ADNI website (www.adni-info.org) and are summarized in section 1.1 in S1 File. MCI subjects met the Petersen (Mayo Clinic) diagnostic criteria for amnestic MCI [32] as follows: (a) a subjective memory complaint; (b) objective memory loss, as measured by age- and education-adjusted scores on Wechsler Memory Scale Logical Memory II, but without significant impairment in other cognitive domains; (c) generally preserved activities of daily living; and (d) no dementia. MCI subjects also had MMSE scores of 24–30 and a global score of 0.5 on the Clinical Dementia Rating (CDR) scale.

From a total of 390 individuals with a baseline diagnosis of MCI who were recruited for ADNI-1, 289 subjects met criteria for inclusion as part of either the P-MCI or N-MCI group in this study. Thirty (~10%) of these subjects were further excluded due to partially missing baseline data. Table 1 shows the characteristics of the MCI subjects included in this study (n = 259). Progressors (P-MCI; n = 139) included MCI subjects who progressed to AD-type dementia within 36 months (median: 18 months) of entering the study, as indicated by the NINCDS-ADRDA criteria for the diagnosis of probable AD [33]. Non-progressors (N-MCI; n = 120) included MCI subjects who had not progressed to dementia within 36 months of entering the study. This group included subjects who remained cognitively stable (n = 107; did not revert to NC status and did not develop dementia) or those who reverted to NC status and remained dementia-free (n = 13).

**Table 1:** Subject characteristics at baseline.

Characteristic	N-MCI (n = 120)	P-MCI (n = 139)	*p*-value
Age, years	74.8 ± 7.6	74.8 ± 7.1	>0.5^a^
Education, years	15.7 ± 2.9	15.6 ± 2.9	>0.5^a^
Sex, % female	28.3	38.1	0.097^b^
APOE ε4 carriers, %	41.7	66.2	<0.001^b^
MMSE score	27.6 ± 1.7	26.7 ± 1.7	<0.001^a^

Values are shown as mean ± standard deviation or percentage. P-values for differences between N-MCI and P-MCI are based on (a) t-test or (b) chi-square test. N-MCI = non-progressive MCI; P-MCI = progressive MCI; APOE = apolipoprotein E; MMSE = Mini-Mental State Examination.

### Data Collection and Follow-up

At study entry (baseline), all subjects underwent a comprehensive clinical evaluation, cognitive/functional assessments, and a structural brain MRI scan. Subjects also provided a blood sample for apolipoprotein E (*APOE*) genotyping and proteomic analysis. Subjects were then followed longitudinally at specific time points (6, 12, 18, 24, 36 months). The clinical status of each MCI subject was re-assessed at each follow-up visit and updated to reflect one of several outcomes (NC, MCI, AD, or other). The N-MCI and P-MCI group designations were based on this follow-up clinical diagnosis and used as the “ground truth” in our classification experiments.

### Clinical Data

We considered a total of 186 clinical variables (features) as potential predictors of MCI-to-dementia progression in our classification analyses. Clinical features were of two types: risk factors (16 features) and assessments/markers (170 features). Risk factors included: age, sex, education, *APOE* genotype, family history of dementia, cerebrovascular disease risk factors, body mass index, and history of psychiatric disorders, alcohol abuse, head trauma, and sleep apnea (see section 1.2 in S1 File). The total scores and sub-scores on the following cognitive, functional, and clinical assessments were considered: Mini-Mental State Examination, Clinical Dementia Rating scale, Functional Activities Questionnaire, Geriatric Depression Scale, Neuropsychiatric Inventory Questionnaire, Modified Hachinski Ischemic Scale, American National Adult Reading Test, WMS-III Logical Memory, Alzheimer's Disease Assessment Scale–Cognitive sub-scale, Rey Auditory Verbal Learning Test, verbal (category) fluency test, Boston Naming Test, digit span test, Trail Making Test, Digit-Symbol Coding Test, and Clock-Drawing Test (see section 1.2 in S1 File for further description). We also included data on whether MCI subjects were on a regimen of AD medications (cholinesterase inhibitors and memantine), a factor shown to be associated with greater cognitive impairment and faster progression to dementia [34]. Recent studies suggest that cognitive and functional markers may be at least as effective as imaging and fluid biomarkers in predicting MCI-to-dementia progression [26,35–37].

### Structural MRI Data

MRI offers a non-invasive, widely available, and more cost-effective alternative for obtaining imaging biomarkers of AD-related neurodegeneration (e.g. atrophy measures) compared to positron emission tomography (PET) [38]. We considered 452 region of interest (ROI)-based morphometric measures computed from individual structural MRI scans as potential predictors of MCI-to-dementia progression. We generated MRI features for classification using an atlas-based ROI method rather than a voxel-based method in an effort to reduce the dimensionality of the MRI dataset and increase the signal-to-noise ratio of the resulting features.

Subjects received high resolution T1-weighted MRI scans of the brain at 1.5 Tesla acquired using a variety of scanners (General Electric, Philips, or Siemens) and a standardized protocol [39]. Each MRI dataset was post-processed using FreeSurfer v5.0.0 (http://surfer.nmr.mgh.harvard.edu) [40–43], an image processing software tool for (a) automated model-based reconstruction and segmentation of the brain's cortical surface and subcortical structures and (b) morphometric analysis. Finally, a variety of morphometric measures were computed across 180 anatomically-defined brain regions as MRI features for classification, including cortical and subcortical volumes, mean cortical thickness (and its standard deviation), surface area, and curvature. FreeSurfer-derived morphometric MRI measures have been validated in studies of normal aging, MCI, and AD [44–46]. See section 1.3 in S1 File for details on MRI acquisition and processing.

### Plasma Proteomic Data

Plasma-based proteomic biomarkers have been proposed as an alternative for the early diagnosis of AD to cerebrospinal fluid (CSF)-based biomarkers [47,48]. However, the utility of plasma biomarkers in predicting MCI-to-dementia progression remains controversial given the conflicting findings in the literature [49,50]. Moreover, at the time of the present study, there were no published reports that utilized the ADNI dataset and pattern classification methods to examine the predictive utility of plasma proteomic biomarkers for predicting MCI-to-dementia progression in combination with clinical and imaging biomarkers (unlike the case with CSF biomarkers). For these reasons, and because blood plasma samples are arguably less invasive and more routinely obtained than CSF samples, we examined plasma proteomic biomarkers as an alternative to CSF biomarkers. Specifically, in addition to clinical and MRI features, we considered 149 features based on plasma protein levels in this study. Plasma samples were analyzed by Rules-Based Medicine (RBM) (Austin, TX) using their Human DiscoveryMAP multiplex immunoassay, which is based on the Luminex xMAP platform [51]. This immunoassay panel of 190 analytes included proteins previously reported to be involved in cell-signaling and/or associated with a variety of disease processes, including AD, metabolic disorders, inflammation, cancer, and cardiovascular disease. The ADNI team, in collaboration with the Biomarkers Consortium, identified 146 (out of 190) analytes that met quality control standards. We used the cleaned, quality-controlled (QC) dataset containing these 146 analytes, labelled “ADNI Plasma QC Multiplex 11Nov2010”. Further details about the RBM immunoassay and QC procedures can be found in the data primer, “Biomarkers Consortium Project: Use of Targeted Multiplex Proteomic Strategies to Identify Plasma-Based Biomarkers in Alzheimer’s Disease” (available at http://adni.loni.usc.edu). We also considered the plasma levels of amyloid-β proteins (Aβ42, Aβ40, and Aβ42/Aβ40 ratio), which were assayed by the ADNI Biomarker Core Laboratory at the University of Pennsylvania. Aβ42 and Aβ40 have been identified as the major molecular species contributing to the amyloid (“senile”) plaques, a pathological hallmark of AD [52].

### Feature Selection and Pattern Classification Approach

Analyses were conducted using MATLAB R2010b (The MathWorks, Inc., Natick, MA). We applied a series of transformations to the feature data prior to conducting feature selection and classification analyses (see section 1.4 in S1 File). Feature selection is a dimensionality reduction strategy that involves identifying a small but informative subset of the original features for classification; it can help avoid model overfitting, improve model performance, and produce models that are easier to interpret and potentially more time- and cost-efficient to develop and use [53]. We adopted a combined filter-wrapper approach to efficiently identify a subset of features that can be used to effectively discriminate between P-MCI and N-MCI. In the “filter” stage, we defined feature subsets of different sizes (ranging from 1 to 50 features) using the Joint Mutual Information (JMI) criterion [54], as implemented in the FEAST toolbox (http://www.cs.man.ac.uk/~gbrown/fstoolbox) [55]. JMI-based feature selection favors features that are maximally relevant to the classification task while being minimally redundant and maximally complementary with previously selected features. In the “wrapper” stage, we evaluated these feature subsets in terms of cross-validated classification accuracy and determined the optimal number of features to be used as a parameter in the final model. Additional details can be found in section 1.5 in S1 File.

In this study, we use the probabilistic multiple kernel learning (pMKL) classification approach proposed by Damoulas et al. (http://www.dcs.gla.ac.uk/inference/pMKL) [30,56,57] to build several prognostic models of dementia. pMKL is a kernel-based classifier similar to the widely used support vector machine (SVM) [20,21]. Kernel classifiers rely on the use of kernel functions to map the original feature data into an inner product space that encodes similarity between examples (e.g. patients). The algorithm learns to classify new examples based on this similarity information. The pMKL classifier, like an SVM, can be used in either the single-kernel mode or the multiple-kernel mode. In the latter case, referred to as multiple kernel learning (MKL), separate kernels are used to encode information from different sources or representations of the data [22]. For further details on the kernels and MKL, see section 1.6 in S1 File. While by design the SVM is a non-probabilistic classifier, the pMKL classifier directly produces probabilistic predictions.

The pMKL classifier is based on a Generalized Linear Model (GLM) regression framework using the multinomial probit likelihood [30] given by:

$$P\;(Y_{n}=i|W,k_{n}^{\beta \;\Theta })=E_{p\;\;(u)}\left\{\prod_{j\neq i}\Phi (u+(w_{i}-w_{j})\;k_{n}^{\beta \;\Theta })\right\}$$

where *E* is the expectation with respect to the standard normal distribution *p*(*u*) = *N*(0,1) and Ф is the cumulative distribution function. This function computes the probability *P* that example *n* belongs to class/outcome *i* (as opposed to class *j*) given the feature data (in the form of a kernel matrix $k_{n}^{\beta \;\Theta }$) and regression coefficients *W*. The regression coefficients reflect the weight with which training examples used to construct the model vote for a particular class/outcome. The posterior probability *P* is determined using Bayesian estimation methods (for details see [57]) and captures the uncertainty or the degree of confidence associated with each prediction. Non-probabilistic classification can be achieved by predicting the class/outcome with the largest posterior probability (>50% for binary classification).

### Experimental Design and Analysis

We built and examined a series of nine predictive models, each designed to classify individual patients as belonging to either the N-MCI or the P-MCI group. Models 1–5 were constructed using a single, linear kernel and were designed to assess the predictive utility of different data sources, alone and in combination. First, a separate single-source model was constructed for clinical risk factors (model 1; 'CRF'), clinical assessments / markers (model 2; 'CAM'), MRI markers (model 3; 'MRI'), and plasma proteomic markers (model 4; 'PPM'). Second, a multi-source model was constructed where all features across the four data sources (CRF, CAM, MRI, and PPM) were concatenated and considered jointly during feature selection and classifier training steps (model 5; 'CONCAT'). We also constructed a set of multiple-kernel, multi-source models (models 6–9) to examine whether multiple kernel learning can be used to improve upon the predictive performance achieved with the single-kernel model (see section 1.7 in S1 File).

In subsequent analyses, we studied the best performing model from the set of nine models examined. First, we examined the extent to which patient heterogeneity affects model accuracy; we examined the effects of age, sex, educational level, *APOE* genotype, presence of cerebrovascular risk factors, off-label use of AD medications, history of depression, and time to progression. Second, we examined the relationship between predictive confidence and model accuracy. Predictive confidence was defined as the difference between the predicted probabilities for the two classes/outcomes (N-MCI and P-MCI). Finally, we examined whether there is an association between the predicted probabilities and time to progression for P-MCI patients.

### Model Performance and Cross-Validation

For each model (1–9), we report several cross-validated measures of predictive performance. We report sensitivity (percent of P-MCI subjects correctly classified) and specificity (percent of N-MCI subjects correctly classified) as measures of classification accuracy [58]. The balanced accuracy rate (BAR), defined as [sensitivity + specificity] / 2, was used as the primary measure of model performance. We also assessed model calibration as a secondary performance measure. Calibration is an important measure of performance for probabilistic classification models and assesses the reliability of the probabilistic predictions [59,60]. The agreement between predicted and actual probabilities (risk of MCI-to-dementia progression) was quantified using the concordance correlation coefficient (CCC; see section 1.8 in S1 File) [61]. Finally, we report the area under the curve (AUC) from the receiver operating characteristic (ROC) analysis as a measure of model discrimination [62].

**Fig 1:**
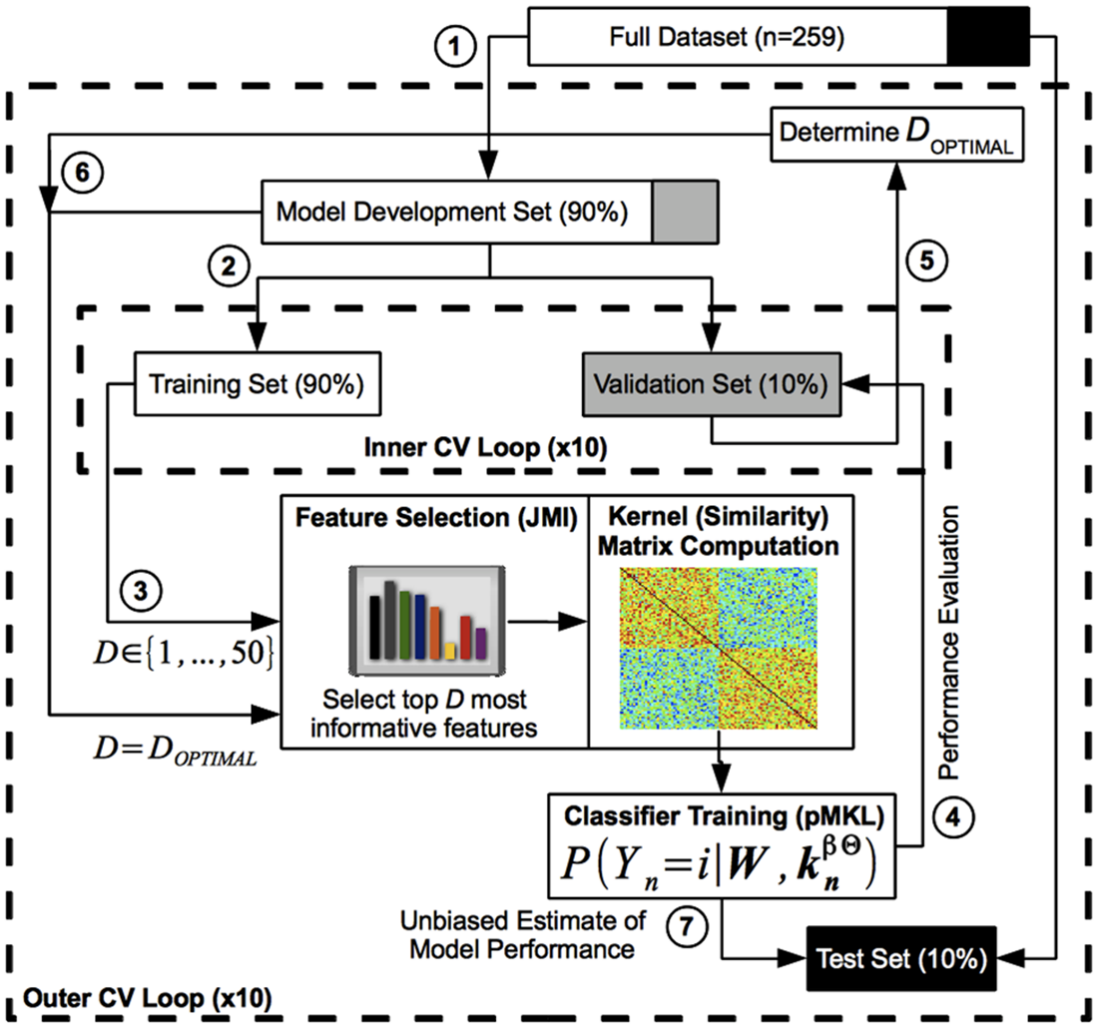
Nested 10-fold cross-validation (CV) procedure for model development and evaluation. (1) In the outer CV loop, the dataset was partitioned into the 'Model Development Set' and 'Test Set'. (2) In the inner CV loop, the 'Model Development Set' was further partitioned into the 'Training Set' and 'Validation Set'. (3) Several classifiers were trained using only the 'Training Set' and a varying number (1–50) of the most informative features, as identified with the Joint Mutual Information (JMI) method. (4) These classifiers were evaluated on the 'Validation Set', and (5) the number of features that produced maximal classification accuracy was selected as the optimal number of features (D_OPTIMAL_). (6) The final model was then constructed by training a classifier using the 'Model Development Set' and the optimal number of JMI-based features, D_OPTIMAL_. (7) An unbiased estimate of model performance was obtained by evaluating the final model on the held out 'Test Set', which was not used during feature selection, model (parameter) selection, or final model construction. Both the outer and inner CV loops used a 10-fold CV design.

We used a nested stratified cross-validation (CV) procedure (Fig 1) to avoid model overfitting and optimistically-biased estimates of model performance [63–65]. The procedure consisted of two nested CV loops, each implementing 10-fold stratified CV: an outer loop, designed to obtain an unbiased estimate of model performance, and an inner loop, designed to select the optimal number of features for the final model (see section 1.9 in S1 File for details). Although during each CV fold the model was developed using data from 90% of the subjects and tested using data from the remaining 10% of the subjects, the model was eventually cross-validated on all 259 subjects. For better replicability, the nested 10-fold CV procedure was repeated 10 times with different partitions of the data, generating 100 performance estimate values for significance testing. We used a modified paired sample *t*-test with 10 degrees of freedom calibrated for 10x10 CV experiments [66] to test for significant differences in performance between model pairs. All statistical tests were considered significant at the *P* < 0.05 level.

## Results

### Predictive Performance of Single-Source and Multi-Source Models

**Fig 2:**
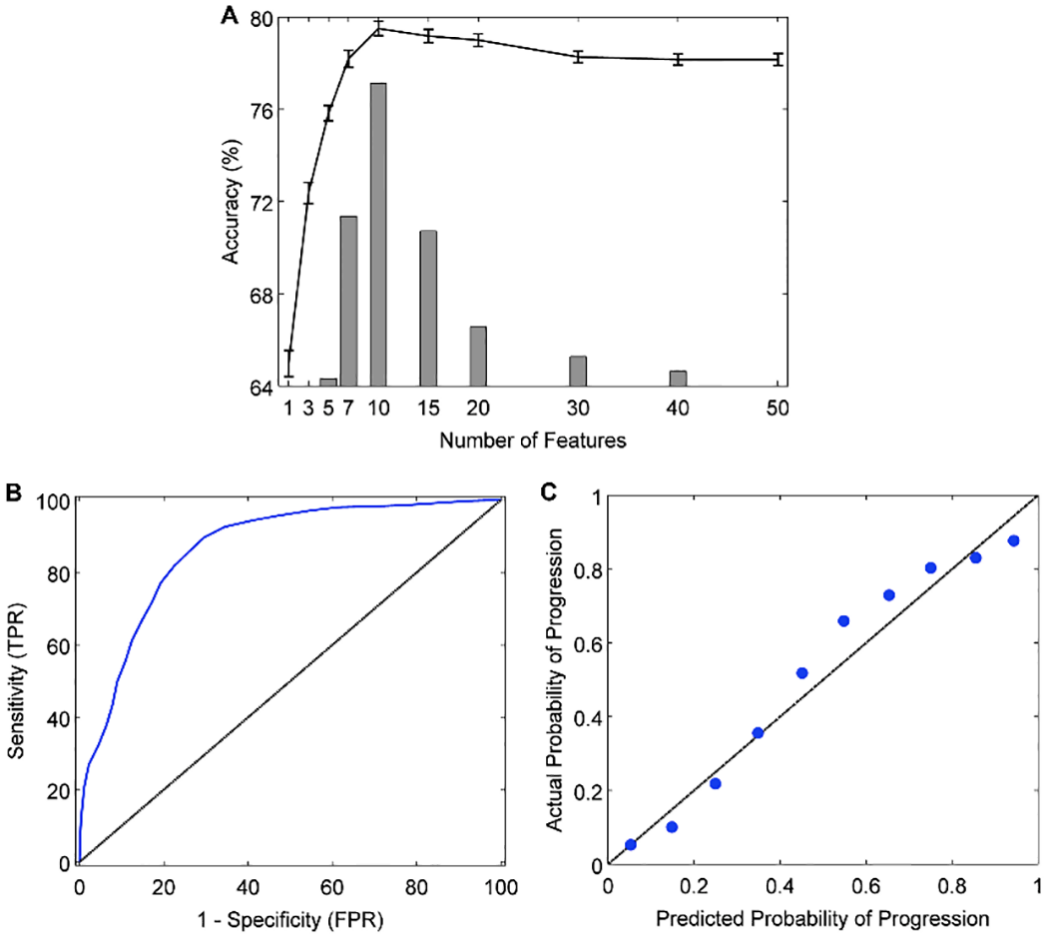
Performance curves for the best performing (MKL-Gaussian) model. A: The learning curve shows validation accuracy as a function of the number of features in the model (line graph with 95% confidence intervals). Juxtaposed is a histogram showing the frequency with which a given number of features was identified as the optimal (most accurate) number of features across 100 trials of the 10x10 cross-validation experiment (median = 10 ± 3). B: Receiver operating characteristic curve (blue line; AUC = 0.87), showing the trade-off between sensitivity (true positive rate, TPR) and 1 –specificity (false positive rate, FPR). The area under the curve (AUC) measures how well the model discriminates between N-MCI and P-MCI patients. The black diagonal line represents random classifier performance (AUC = 0.5). C: Calibration curve, indicating the degree to which the model's predicted probabilities (risk) of MCI-to-dementia progression agree with the actual probabilities of progression. With a perfectly calibrated model, we expect complete agreement between predicted and actual probabilities (diagonal line).

Table 2 and S1 Table summarize the predictive performance of models 1–9. Validation and test set accuracies (V-BAR and T-BAR) were within 3% of each other for all models, and in many cases <1% apart, indicating that model overfitting was minimal and that our nested cross-validation procedure was effective. We compared the various models in terms of their classification accuracy (indicated by the balanced accuracy rate on the test set, T-BAR) and calibration (indicated by the CCC). The accuracies of all four single-source models (1–4: CRF, CAM, MRI, PPM) exceeded chance-level (all *P* < 0.01, one-sample *t*-test), although they varied from a low of 53.2% for PPM to a high of 76.1% for CAM. The CAM model outperformed the other three single-source models on accuracy (all *P* < 0.001, paired-sample *t*-test). The CAM and MRI models were well-calibrated, as indicated by high positive CCC (both *P* < 0.001) while the PPM model showed poor calibration (CCC not different from zero, *P* > 0.3). The single-kernel, multi-source model 5 (CONCAT), in which all features across the four data sources were considered jointly, outperformed all four single-source models (all *P* < 0.001, paired-sample *t*-test) with an accuracy of 80.0%. The calibration of the CONCAT model, as measured by the CCC, was statistically similar to that of CAM and MRI models (both *P* > 0.3) and better than that of the PPM model (*P* < 0.001). None of the four multiple-kernel, multi-source models considered (models 6–9) outperformed the single-kernel CONCAT model in terms of classification accuracy. However, model 6 ('MKL-Gaussian', a multi-source model constructed using 5 Gaussian kernels) outperformed the single-kernel CONCAT model in terms of calibration, as indicated by a higher CCC (*P <* 0.05), while maintaining a similar accuracy of 79.9%. Based on its classification accuracy and calibration, model 6 (MKL-Gaussian) was selected as the best performing model to be studied in subsequent analyses. Fig 2 shows the learning, ROC, and calibration curves that further characterize the predictive performance of the MKL-Gaussian model. In the case of our best performing model (MKL-Gaussian), a median of 10 ± 3 features were selected as predictors of MCI-to-dementia progression.

**Table 2:** Cross-validated performance estimates for single-source and multi-source models.

Model (#)	V-BAR (%)	T-BAR (%)	Sn (%)	Sp (%)	AUC-ROC	CCC	*D*_OPTIMAL_ / Total
*Single Source*							
CRF (1)	62.0 ± 1.4	61.8 ± 7.7	65.3 ± 12.7	58.3 ± 11.7	0.61 ± 0.12	#	1 ± 0 / 16
CAM (2)	77.9 ± 1.4	76.1 ± 7.2	76.9 ± 9.5	75.3 ± 11.2	0.83 ± 0.07	0.92 ± 0.03	15 ± 10 / 170
MRI (3)	71.4 ± 1.6	69.1 ± 8.5	68.5 ± 11.8	69.6 ± 12.4	0.76 ± 0.09	0.91 ± 0.03	10 ± 5 / 452
PPM (4)	56.0 ± 2.7	53.2 ± 10.0	51.2 ± 12.9	55.3 ± 14.1	0.54 ± 0.11	0.10 ± 0.31	40 ± 10 / 149
*Multi-Source*							
CONCAT (5)	79.7 ± 1.4	80.0 ± 7.3	80.3 ± 10.6	79.8 ± 10.9	0.86 ± 0.07	0.93 ± 0.02	10 ± 3 / 787
MKL-Gaussian (6)	80.3 ± 1.3	79.9 ± 6.8	83.4 ± 9.9	76.4 ± 12.3	0.87 ± 0.07	0.95 ± 0.01	10 ± 3 / 787

For each model, several measures of predictive performance are shown (mean ± standard deviation), including balanced accuracy rate on the validation set (V-BAR) and the test set (T-BAR), sensitivity (Sn), specificity (Sp), area under the curve (AUC), and concordance correlation coefficient (CCC). D_OPTIMAL_ is the optimal number of features (shown as median ± median absolute deviation); this parameter was determined via cross-validation (see text). The total number of potential features considered when building each model is shown for reference. Performance estimates for models 7–9 are shown in S1 Table. CRF = Clinical Risk Factors, CAM = Clinical Assessments/Markers, MRI = Magnetic Resonance Imaging, PPM = Plasma Proteomic Markers. Models 1–4: single linear kernel using features only from the given data source (CRF, CAM, MRI, PPM). Model 5 (CONCAT): single linear kernel, concatenating features from all data sources. Model 6 (MKL-Gaussian): 5 Gaussian kernels using features from all data sources. # Robust estimate of CCC could not be obtained for model 1 because only <10 probability sub-intervals could be defined when conducting calibration analysis.

### Predictors of MCI-to-Dementia Progression

**Fig 3:**
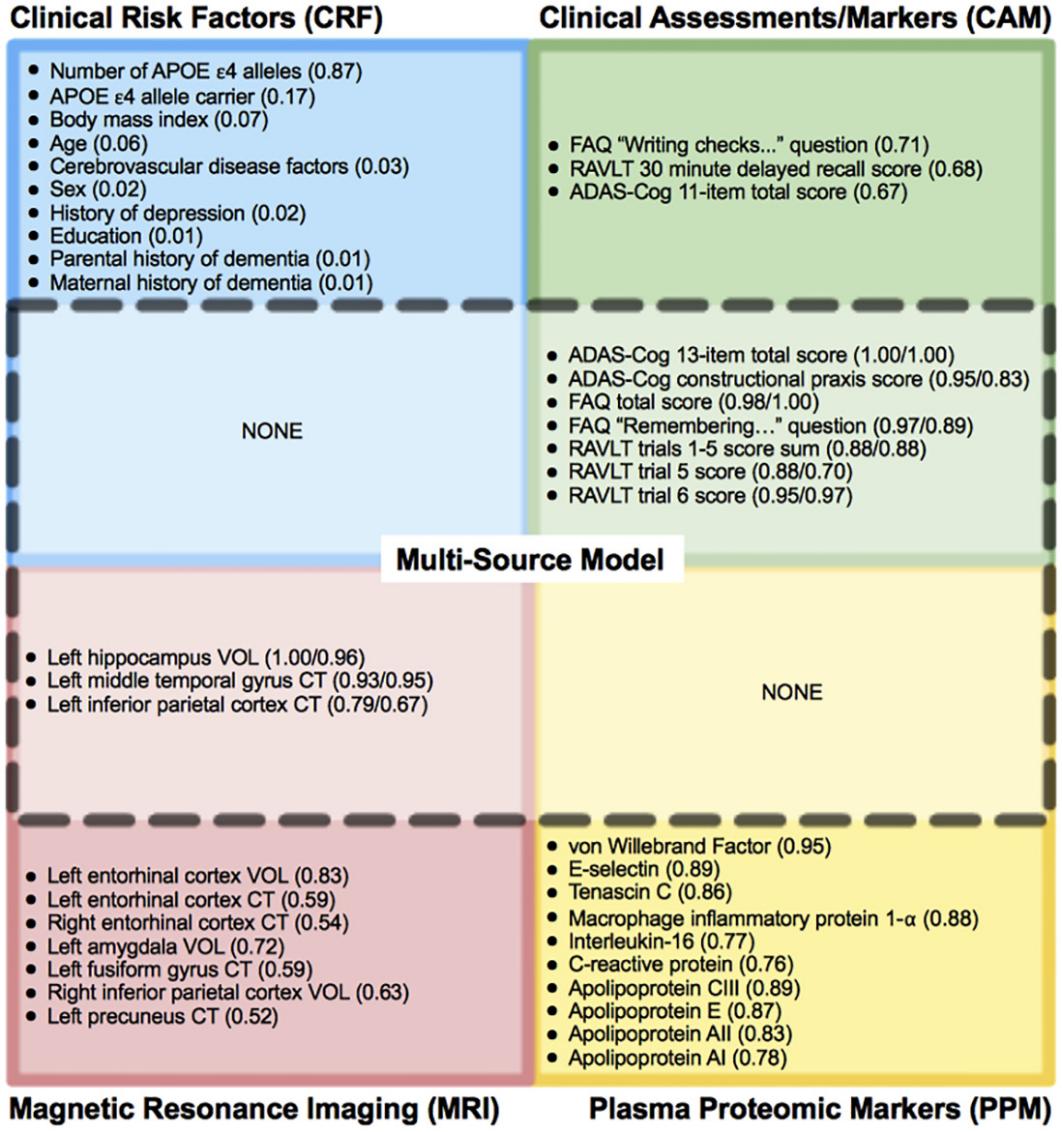
Top 10 most frequently selected features as baseline predictors of MCI-to-dementia progression. Features are shown separately for each single-source model: CRF (blue), CAM (green), MRI (red), PPM (yellow). A subset of these features (dashed line) was selected as part of both the single-kernel (CONCAT) and the multiple-kernel (MKL-Gaussian) multi-source models and included only CAM and MRI features. The selection frequency across 100 trials of the 10x10 cross-validation experiment is shown in parentheses as: (#) for single-source model only or (#/#) for both single/multi-source (MKL-Gaussian) models. APOE = apolipoprotein E, ADAS-Cog = Alzheimer's Disease Assessment Scale–Cognitive sub-scale, FAQ = Functional Activities Questionnaire, RAVLT = Rey Auditory Verbal Learning Test, VOL = volume, CT = cortical thickness.

**Fig 4:**
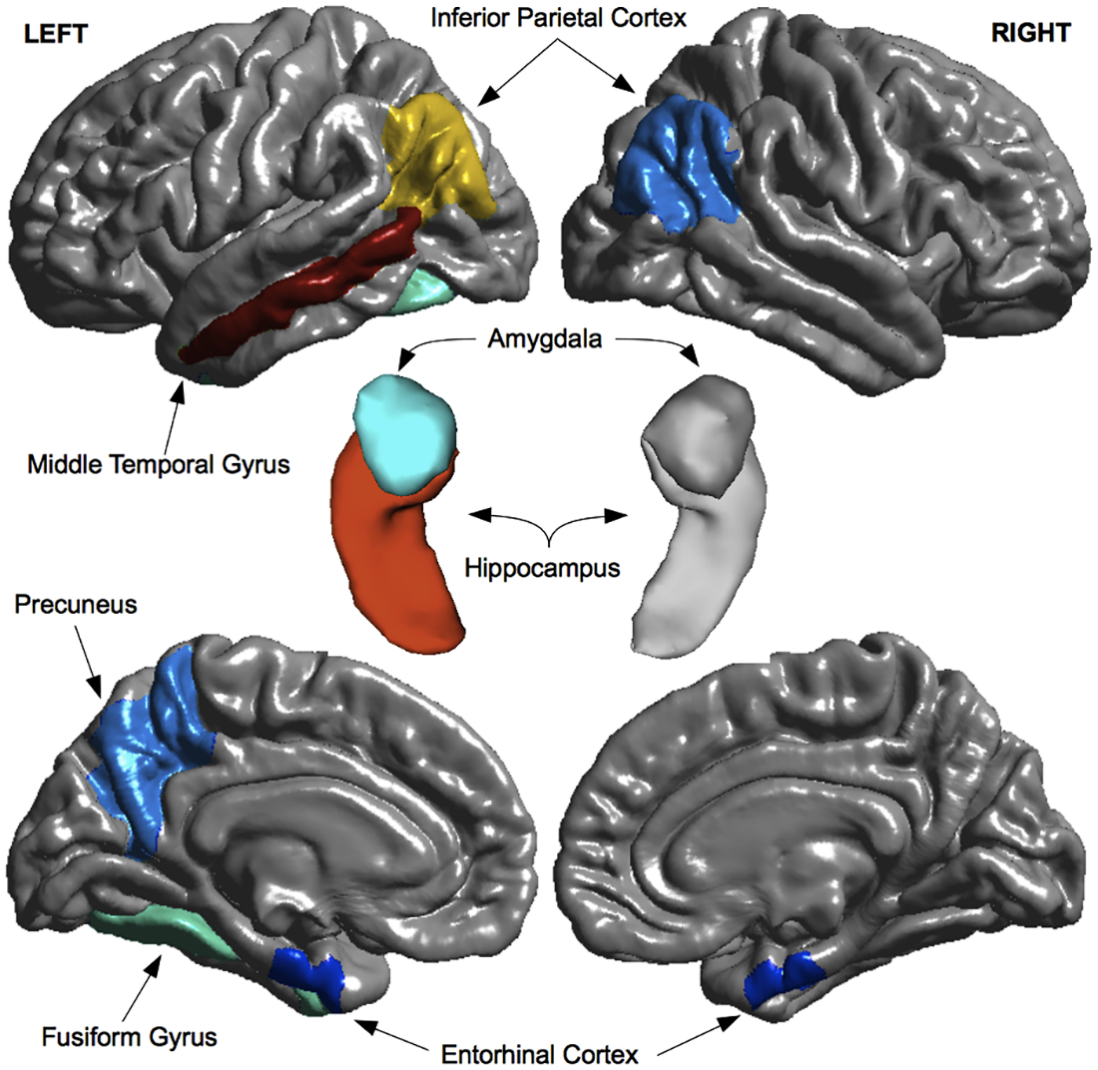
Regional MRI predictors of MCI-to-dementia progression. Morphometric measures (volumes and cortical thickness) for brain regions shown in both warm and cool colors were selected as predictors in the single-source MRI model. Morphometric measures for a subset of these regions, shown in warm colors (red, orange, yellow), were also selected as predictors in multi-source (CONCAT and MKL-Gaussian) models. Regions of interest are overlaid on top of 3-D model reconstructions of the brain (gray). Top row: lateral view of the cerebral hemispheres. Center: close-up view of the hippocampus-amygdala complex. Bottom row: medial view of the cerebral hemispheres.

Fig 3 shows the top 10 features that were most frequently selected as baseline predictors of MCI-to-dementia progression for each of the single-source models (CRF, CAM, MRI, PPM) and two multi-source models (CONCAT and MKL-Gaussian). Fig 4 shows the topography of the brain regions selected as predictors in the single-source MRI model and the multi-source models (CONCAT and MKL-Gaussian). Among the features considered for selection in the CRF model, only the number of *APOE* epsilon 4 alleles was selected with a high degree of consistency. Other candidate CRF features, including age, were selected infrequently. The features most frequently selected in the CAM model included total scores and sub-scores on three assessments: Alzheimer's Disease Assessment Scale–Cognitive sub-scale (ADAS-Cog), Functional Activities Questionnaire (FAQ), and Rey Auditory Verbal Learning Test (RAVLT). In the MRI model, the most frequently selected features included volume and cortical thickness measures for several temporoparietal brain regions with a preference toward the left hemisphere (8/10 features). In the PPM model, the most frequently selected features included proteins associated with vascular processes, immune function and inflammation, and lipid metabolism. In the case of multi-source models–both the single-kernel (CONCAT) model and the best performing, multiple-kernel (MKL-Gaussian) model–only CAM and MRI features were consistently selected as predictors (Fig 3). CAM predictors included the 13-item total score and constructional praxis sub-score on the ADAS-Cog, the total score and memory question sub-score on the FAQ, as well as the sum of scores across trials 1–5, trial 5 sub-score, and trial 6 sub-score on the RAVLT. MRI predictors included left hippocampal volume, left middle temporal cortical thickness, and left inferior parietal cortical thickness (Fig 4).

**Fig 5:**
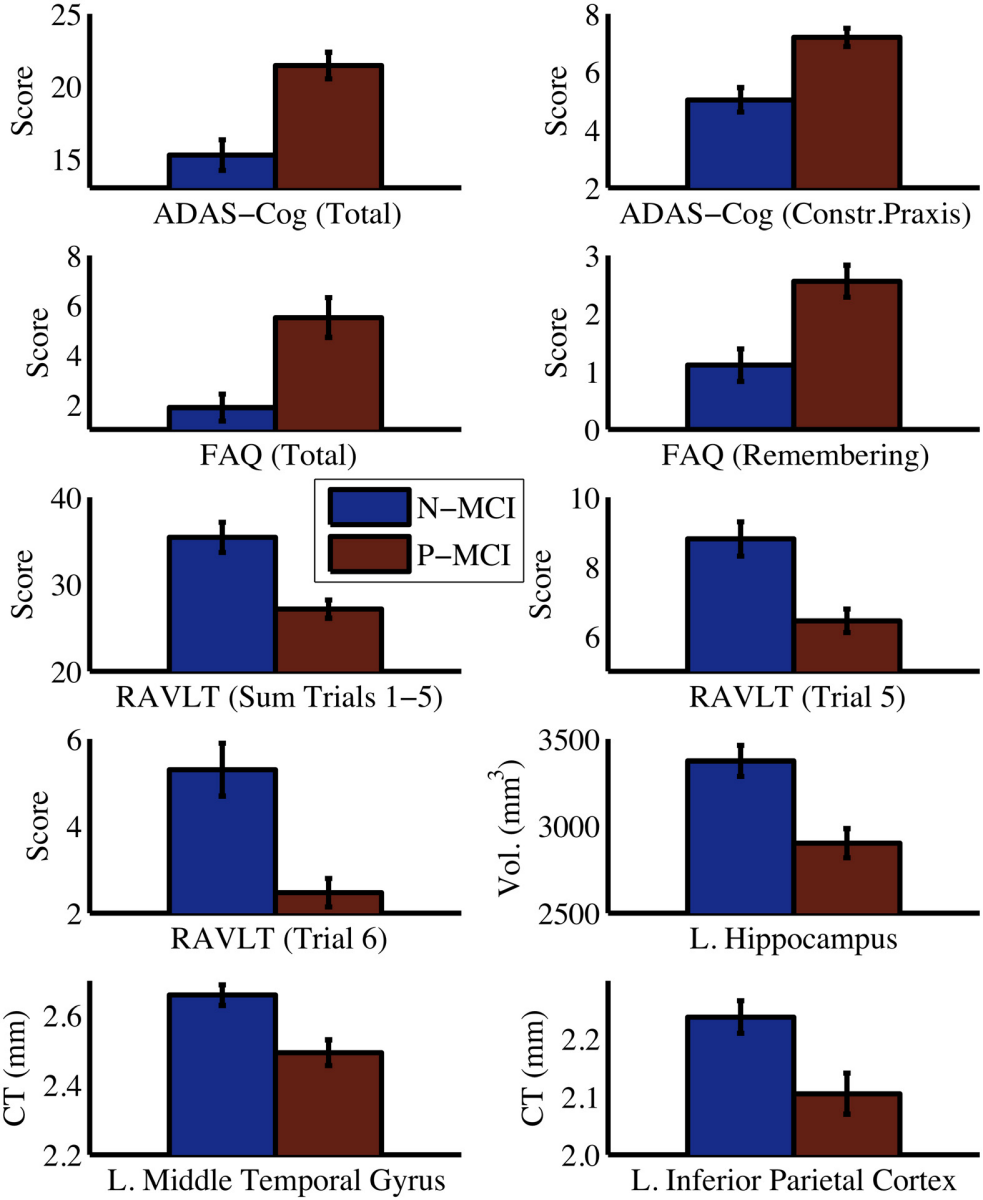
Comparison between N-MCI and P-MCI groups on baseline predictor variables. Error bars are 95% confidence intervals. Significant group differences were present for all predictor variables (all P < 0.001). Vol. = volume, CT = cortical thickness, ADAS-Cog = Alzheimer's Disease Assessment Scale–Cognitive sub-scale, FAQ = Functional Activities Questionnaire, RAVLT = Rey Auditory Verbal Learning Test, L. = Left, Constr. = Constructional

As a confirmatory analysis, we compared N-MCI and P-MCI groups on each of the baseline predictors identified in the multi-source models (Fig 5). As expected, there was a robust statistically significant difference between the two MCI groups for all predictor variables (all *P* < 0.001, independent sample *t*-test). P-MCI subjects were more cognitively and functionally impaired at baseline than N-MCI subjects, as indicated by higher scores on the ADAS-Cog and FAQ. Relative to N-MCI subjects, P-MCI subjects had a more pronounced verbal memory impairment at baseline, as indicated by lower scores on the RAVLT. P-MCI subjects also showed signs of atrophy in temporoparietal brain regions at baseline, as indicated by reduced hippocampal volume as well as reduced middle temporal and inferior parietal cortical thickness relative to N-MCI subjects. In this study, we excluded data from 131 out of 390 (~34%) MCI subjects in the ADNI-1 database because they either did not meet our inclusion criteria or due to missing data. No differences were found between included (n = 259) and excluded (n = 131) subjects on any of the baseline predictor variables (S1 Fig), suggesting that a selection bias is unlikely to have been introduced due to our exclusion of subjects.

### Effect of Patient Characteristics on Model Performance

**Fig 6:**
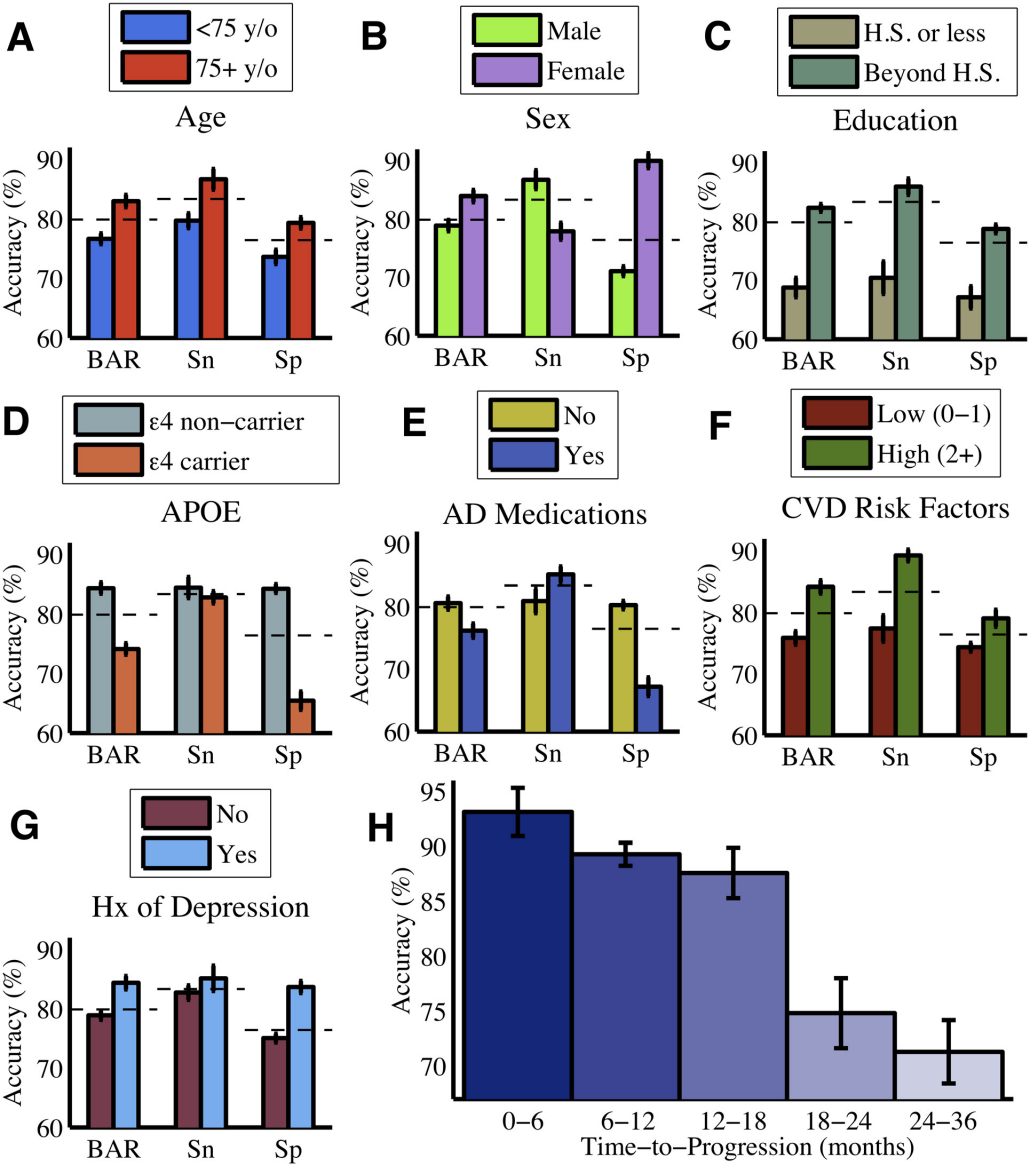
Effect of patient characteristics on classification accuracy. The classification accuracy of the model (MKL-Gaussian) varied with baseline demographic (A-C), genetic (D), and clinical (E-G) characteristics. Panel E compares MCI patients who were on a regimen of AD medications versus those who were not. Panel F compares patients according to the number of pre-existing conditions in their medical history that are considered to be cerebrovascular disease (CVD) risk factors, including diabetes mellitus, coronary artery disease, hypertension, smoking, hyperlipidemia, and stroke. The classification accuracy of the model varied inversely with time to progression for P-MCI patients (H). The overall accuracy of the model (as found in Table 2) is shown for reference as a dashed line. Error bars represent 95% confidence intervals across cross-validation trials. BAR = Balanced Accuracy Rate, Sn = Sensitivity, Sp = Specificity, y/o = years old, H.S. = high school, Hx = history, APOE = apolipoprotein E, AD = Alzheimer's disease.

Further analysis of the best performing (MKL-Gaussian) model revealed that overall the model generated more accurate predictions regarding MCI-to-dementia progression for subjects with the following characteristics (Fig 6A–6G): older age; females; higher educational level; *APOE* epsilon 4 allele non-carriers; not using AD medications; multiple cerebrovascular disease risk factors; or a history of depression. The particular effects on sensitivity and specificity were more variable. In the case of P-MCI subjects, classification accuracy was inversely related to the time to progression from MCI to dementia (Fig 6H): 0–6 months (93.1%), 6–12 months (89.3%), 12–18 months (87.6%), 18–24 months (74.8%), 24–36 months (71.3%). MCI-to-dementia progression could be predicted with substantially greater accuracy if it occurred within the first 18 months after baseline (89.4%) rather than during months 18–36 (73.3%).

### Predictive Confidence and Accuracy

**Fig 7:**
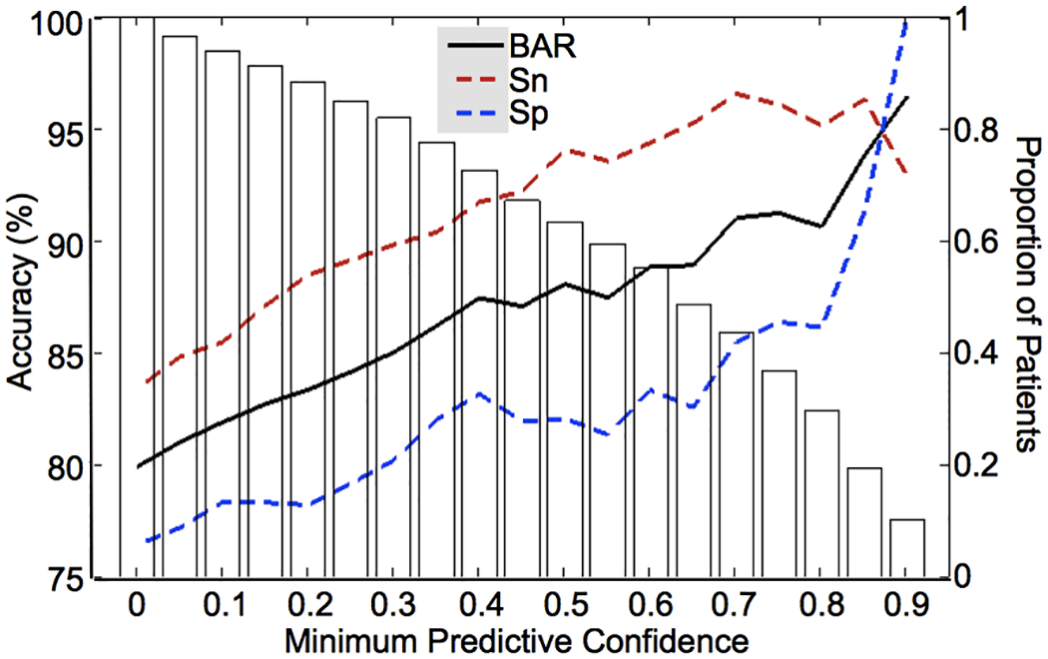
Model accuracy as a function of predictive confidence. Increasing the minimum confidence required to make predictions resulted in improved model accuracy (solid and dashed lines; left y-axis), albeit at the cost of a decreasing proportion of MCI patients for whom "high confidence" predictions could be made (white bars; right y-axis). Predictive confidence was defined as the difference between the predicted probabilities for the N-MCI and P-MCI groups. BAR = Balanced Accuracy Rate, Sn = Sensitivity, Sp = Specificity.

We investigated whether probabilistic outputs from the pMKL classifier could be used to improve the classification accuracy of our prognostic model by permitting only "high confidence” predictions to be made. As we raised the level of confidence required to make predictions, the accuracy of the model gradually increased (Fig 7). However, this increase in classification accuracy came at a cost; with increasing minimum level of confidence required, the model was able to make such "high confidence" predictions for an increasingly smaller proportion of patients. For example, requiring a minimum predictive confidence level of 0.4 (corresponding to predicted probabilities of 0.70 for P-MCI and 0.30 for N-MCI or vice versa), improved model accuracy from 79.9% (83.4% sensitivity, 76.4% specificity) to 87.4% (91.7% sensitivity, 83.2% specificity). This improved accuracy was achieved by allowing predictions to be made only for the top ~73% most confident patient cases, while designating the predictions for the other ~27% of patient cases as “ambiguous” or “low confidence”. We also examined whether probabilistic outputs from the pMKL classifier reflect the time to progression information for individual P-MCI subjects. Correlation analysis revealed that there was a small but statistically significant negative association between the predicted probability (risk) of progression and the time to progression (i.e. larger probability of progression was associated with shorter time to progression; r = -0.20, *P* < 0.05, Spearman correlation).

## Discussion

### Predictive Utility of Clinical, MRI, and Plasma Proteomic Data

Cognitive and functional (CF) assessments proved to be the most accurate (76.1%) in predicting MCI-to-dementia progression. Likewise, other studies have reported that CF markers are more predictive of MCI-to-dementia progression than structural MRI and CSF biomarkers during a two-year period [35,26]. Plasma proteomic data had the lowest predictive accuracy (53.2%), which was only marginally better than chance. In addition, the median number of plasma proteomic features selected as predictors was substantially larger than that for other data sources (40 versus 15 or less). This suggests that as a potential source of biomarkers, plasma proteomic data have a low signal-to-noise ratio and limited utility for predicting MCI-to-dementia progression over a three-year period. Using a different pattern classification strategy, Johnstone and colleagues [50] also found that plasma-based proteomic measures could not reliably discriminate between P-MCI and N-MCI subjects. The predictive accuracy of MRI measures (69.1%) and clinical risk factors (61.8%) was found to be intermediate between that of CF assessments and plasma proteomic measures. Multi-source models (CONCAT and MKL-Gaussian) yielded an improvement in predictive accuracy up to ~80%–beyond that achieved with any single source of data alone. In these more accurate models, only CF assessment scores and morphometric MRI measures were consistently identified as predictors, indicating that these data sources provide complementary information regarding MCI-to-dementia progression. In contrast, clinical risk factors and plasma proteomic measures were not consistently selected as predictors, indicating that these data sources provide limited or redundant information about progression.

Interestingly, we found that our best performing model (MKL-Gaussian) could identify MCI patients who progressed to AD dementia within 18 months of baseline with substantially higher accuracy than patients who progressed after 18 months. Thus, CF and MRI markers appear to be most sensitive to incipient AD during the 18 months prior to the onset of dementia. This finding is consistent with the AD biomarker model proposed by Jack and colleagues [67], which states that different biomarkers have unique temporal trajectories and may be optimally sensitive to AD-related changes during specific time periods. While clinical measures and markers of neuronal injury (e.g. MRI-based atrophy) become abnormal during later stages of AD and may be useful for predicting short-term progression, markers of amyloid deposition become abnormal early and may be more useful for predicting long-term progression.

### Cognitive, Functional, and MRI Predictors

The predictors of MCI-to-dementia progression identified in the multi-source models included baseline scores on cognitive (ADAS-Cog and RAVLT) and functional (FAQ) assessments as well as morphometric measures for three brain regions (left hippocampus, middle temporal gyrus, and inferior parietal cortex). The selection of ADAS-Cog scores as predictors, in addition to RAVLT scores, suggests that baseline impairment in multiple cognitive domains–not just memory function–is predictive of future progression to dementia. Consistent with this finding, previous studies have reported that MCI patients with both memory and non-memory deficits have a greater risk of progression to AD dementia than those with isolated memory deficits [68]. A large meta-analysis also concluded that impairments across multiple cognitive domains are evident several years prior to the clinical diagnosis of AD-type dementia [69]. Alternatively, impairment in multiple cognitive domains, as measured by performance on the ADAS-Cog, can be viewed as reflecting a more advanced MCI stage. In this “late” MCI stage, the patient is further along the normal-MCI-dementia continuum and closer to crossing the clinical threshold from MCI to dementia. The selection of FAQ scores as predictors indicates that a subtle but reliable impairment in functional status precedes the development of overt dementia in patients with MCI. This finding challenges one of the principal distinctions between MCI (as defined by the original Petersen/Mayo Clinic criteria) and dementia–whether the ability to perform activities of daily living is preserved [32].

It is important to note that in this study, MCI subjects recruited as part of ADNI-1 were diagnosed based on the original Petersen (Mayo Clinic) criteria for amnestic MCI [32]. Thus, MCI subjects were limited to those with memory-only impairments (without significant impairments in other cognitive domains), also termed single-domain amnestic MCI, and those with preserved activities of daily living. Nevertheless, our results suggest that, even among these MCI patients diagnosed using the single-domain amnestic MCI definition, subtle impairments in both cognitive domains in addition to memory and in functional status were predictive of MCI-to-AD progression. Importantly, our results provide empirical support to the most recently revised clinical criteria for MCI, where the concept of “MCI due to AD” is proposed to include “impairment in one or more cognitive domains” and an allowance for “mild problems performing complex functional tasks” [70].

The selection of hippocampus, middle temporal gyrus, and inferior parietal cortex as predictors of MCI-to-dementia progression is consistent with the known pattern of grey matter atrophy associated with incipient AD, which begins in the medial temporal lobes and then spreads to temporoparietal association cortices [71]. In both the single-source MRI and multi-source models, morphometric MRI features were selected as predictors with a preference toward the left hemisphere, consistent with evidence that AD-related atrophy occurs at a faster rate in the left hemisphere [71].

### Effect of Multiple Kernel Learning (MKL) on Model Performance

The effect of MKL on model performance was modest in this study. MKL did not improve classification accuracy but modestly improved the calibration of the multi-source model when using five Gaussian kernels. We used a relatively small number (3–5) of kernels in our MKL models, which could account for the limited benefit we observed with MKL. Using a larger number of kernels, as done in some recent studies (e.g. [23]), could yield additional improvements in predictive performance.

### Comparison with Models in the Literature

Table 3 shows that our best prediction model (AUC = 0.87, accuracy = 79.9%) performed very favorably compared with recently published models. For better compatibility with the present study, we limit this comparison to studies that used baseline data from the ADNI dataset to predict MCI-to-AD progression within a 24–48 month follow-up period. By incorporating CF markers along with other biomarkers (as done in our study), recent studies have achieved AUCs in the 0.80–0.87 range. Gomar et al. [35] attained an AUC of 0.80 by combining CF and MRI markers in a logistic regression model. Cui et al. [26] also attained an AUC of 0.80 by combining CF, MRI, and CSF markers using an SVM classifier, although they trained their model on data from healthy control and AD subjects rather than on MCI data as we did. Ye et al. [72] developed an SVM-based model that included CF and MRI markers as well as *APOE* genotype, obtaining an AUC of 0.86. Devanand et al. [73] proposed a logistic regression model that incorporated CF and MRI markers and had an AUC of 0.87 (77% accuracy), although the predictive accuracy of this model was reported to be higher (85%) in an earlier and smaller, single-center, non-ADNI study [74].

**Table 3:** Comparison of models for predicting MCI-to-AD progression.

Study	Time (months)	Markers	AUC-ROC	Acc (%)	Sn (%)	Sp (%)
*Present study*	36	CF, MRI	0.87	79.9	83.4	76.4
Cui et al. (2011)	24	CF, MRI, CSF	0.80	67.1	96.4	48.3
Gomar et al. (2011)	24	CF, MRI	0.80	71.9	56	82
Hinrichs et al. (2011)	36	MRI, PET	0.74	—	—	—
Westman et al. (2012)	36	MRI, CSF	0.76	68.5	74.1	63.0
Ye et al. (2012)	48	CF, MRI, APOE	0.86	—	—	—
Zhang and Shen (2012)	24	MRI, PET, CSF	0.80	73.9	68.6	73.6
Wee et al. (2013)	36	MRI	0.84	75.1	63.5	84.4
Young et al. (2013)	36	MRI, PET, APOE	0.80	74.1	78.7	65.6

AUC = area under the curve, Acc = accuracy, Sn = sensitivity, Sp = specificity, CF = cognitive/functional markers, MRI = magnetic resonance imaging, CSF = cerebrospinal fluid, PET = positron emission tomography, APOE = apolipoprotein E genotype.

Other recent studies using the ADNI dataset have developed models based on various combinations of MRI, PET, and CSF markers, attaining AUCs in the 0.74–0.80 range [23,24,28,75]. Similar to our study, Young et al. [75] also used a probabilistic kernel-based classification approach for predicting MCI-to-AD progression. Their best performing model incorporated MRI, PET, and *APOE* markers and had an AUC of 0.80 (74.1% accuracy). Two methodological differences may account for the superior predictive accuracy of our model compared to that of Young et al. Unlike their model, our model incorporated CF markers. Moreover, while their model was trained on data from healthy control and AD subjects (and then used to classify MCI subjects), we trained our model using MCI data to specifically classify N-MCI and P-MCI subjects. Using a model that incorporated only baseline MRI data, Wee et al. [76] were able to predict MCI-to-AD progression with surprisingly high accuracy (AUC = 0.84). Although we achieved better accuracy with our multi-source model, the MRI model of Wee et al. significantly outperformed our single-source MRI model (AUC = 0.76). In addition to using ROI-based morphometric features (as we did in our study), they also used correlational features that captured the inter-regional similarity in cortical thickness, potentially providing a way to improve our prediction model in the future. Finally, the predictive accuracy of our model was not only high but also fairly balanced with a sensitivity/specificity differential of only 7%, which compares favorably with recent studies where this differential was as high as 48%.

### Importance of Patient Heterogeneity

We found that the predictive accuracy of our multi-source model (MKL-Gaussian) varied with demographic, genetic, and clinical characteristics even though none of these variables were selected as predictors of progression. For example, accuracy tended to be higher when classifying older MCI patients but lower for carriers of the *APOE* epsilon 4 allele. A possible mechanism for this interaction between predictive accuracy and patient characteristics is that variables such as age and *APOE* genotype may be exerting moderator effects on the CF and MRI predictors in our model. Supporting this explanation is evidence that aging and AD exert independent but partially overlapping effects on cognitive function and brain structure, including converging effects on the hippocampus and temporoparietal cortex [77,78]. In the case of *APOE* genotype, epsilon 4 allele has been linked with temporal lobe atrophy, an effect seen even in healthy control subjects [79]. We did not explicitly account for such potential moderator effects in our classification analyses, as this was beyond the scope of our study. However, we did consider patient characteristics such as age and *APOE* genotype as predictors of progression and examined their interaction with other predictor variables insofar as these interactions were identified using the JMI-based multivariate feature selection technique used in this study. Future studies may be able to improve predictive accuracy further by removing moderator-related variability from the data via stratification or regression methods [80]. Nevertheless, our findings suggest that it is important to consider the effects of patient heterogeneity when developing predictive models of dementia. It is not safe to assume that a model performs equally well across different strata of the patient population. An analysis of predictive accuracy stratified according to various patient characteristics could identify if the model performs poorly for specific subgroups of individuals and highlight areas for improvement.

### Probabilistic Classification of MCI: Advantages and Applications

A unique aspect of this study is our adoption of a probabilistic kernel-based classifier (pMKL) for the prediction of MCI-to-dementia progression. Calibration analysis revealed that the probabilistic predictions generated by our model reliably reflect the actual risk of progression. Thus, the model could be used to stratify MCI patients according to the risk of progression. The probabilistic predictions also reflected some information about the time to progression for P-MCI patients, a surprising finding since the model was not explicitly trained to predict time to progression but rather to classify P-MCI versus N-MCI subjects. It may be possible to adapt our pattern classification approach to explicitly predict time to progression, which would allow staging of MCI patients along the MCI-AD continuum.

Importantly, we showed that the probabilistic outputs could be used as a measure of predictive confidence to further improve the accuracy of the model. When using the model in conventional, non-probabilistic mode, where no information about predictive confidence was taken into account, we obtained an accuracy of 79.9% (83.4% sensitivity, 76.4% specificity). When using the model in probabilistic mode, where predictions were allowed to be made only for the top ~73% most confident patient cases, we obtained an improved accuracy of 87.4% (91.7% sensitivity, 83.2% specificity). By assuming a 30% risk of progression over a three-year period (~10% annually) as the pre-test probability [3,6], we obtain positive post-test probabilities of 60.2% (non-probabilistic) and 70.1% (probabilistic) and a negative post-test probabilities of 8.5% (non-probabilistic) and 4.1% (probabilistic) via application of Bayes' rule ([29]. This means that 60.2% (non-probabilistic) and 70.1% (probabilistic) of amnestic MCI patients that our model designates as “progressors” would progress to dementia within a three-year period. Conversely, only 8.5% (non-probabilistic) and 4.1% (probabilistic) of patients that our model designates as “non-progressors” would progress to dementia within a three-year period.

Our probabilistic prognostic model could be used to stratify MCI patients into high and low risk groups as a way to enrich a patient sample in a clinical trial, resulting in up to a 57% reduction in the required sample size to detect the effect of a potential treatment. The extent of amyloid deposition in the brain based on CSF proteomic analysis or PET imaging is already being used as a biomarker to select “amyloid positive” individuals in clinical trials. For example, the ongoing A4 clinical trial (Anti-Amyloid Treatment in Asymptomatic Alzheimer’s Disease) is designed to examine whether an anti-amyloid treatment can slow down cognitive decline among non-demented older adults who have amyloid deposition in their brains, as determined using PET-based amyloid scans [81]. Our predictive model offers an alternative approach for selection of individuals at risk for developing AD in clinical trials. The model could also be used to more accurately identify high-risk MCI patients for early treatment with disease-modifying agents. In cases where the model cannot make a confident prediction, the clinician can then choose to order additional biomarker tests. Through the use of our prognostic model, more expensive, more invasive, or less widely available tests (e.g. PET-based amyloid imaging) could thus be used more sparingly, to the great benefit of the healthcare economy and the patients.

### Limitations and Future Directions

An inherent limitation of this and other pattern classification studies using the ADNI dataset is the reliance on the clinical diagnosis of AD as the "ground truth" (gold standard). The clinical diagnosis of probable AD has an accuracy of 70–90% relative to the pathological diagnosis [82] The implication of this is that models developed to predict progression from MCI to clinically-diagnosed AD can only be as accurate as the clinical diagnosis itself. Also, the relative uncertainty of the clinical diagnosis means that additional variability (noise) is introduced into the model development process, making the prediction task more challenging. Furthermore, the use of clinical criteria to identify when MCI-to-AD progression occurs may in part explain why baseline clinical assessments–which capture similar information–are often more predictive of progression than other types of biomarkers. We recognize that there may be potential concern about circular reasoning when using clinical assessment scores as predictors of MCI-to-dementia progression. However, we believe the prospective nature of the clinical assessments as predictors in the present study (which were collected 6–36 months prior to the clinical outcome of interest) substantially mitigates this concern. To further address these issues, future research on predictive models of AD should incorporate not only data from clinically-diagnosed patients but also from those diagnosed using established pathological criteria.

Another limitation of this study is the relatively short follow-up period of three years. Although the development of prognostic models for long-term dementia prediction is warranted, short-term dementia prediction can be useful for selecting high-risk MCI patients in clinical trials. For example, some recent clinical trials investigating disease-modifying anti-amyloid agents for the treatment (e.g. [83]) or prevention (e.g. [81]) of AD have been 1.5 and 3 years in duration, respectively. It is also important to note that the majority of MCI patients who subsequently develop AD-type dementia do so within the first few years of follow-up [84]. Finally, although in this study we considered only the *APOE* genotype as a generic predictor of progression, genome-wide association studies have been used to identify several other genes that likely contribute to the development of AD [85]. These AD-related susceptibility genes should be investigated in future work to determine their utility in predicting MCI-to-dementia progression.

The present work can be extended in several ways. First, our model was developed using data only from patients with the single-domain amnestic subtype of MCI, based on the inclusion criteria of ADNI-1. Thus, the use of this relatively narrow inclusion criteria means that multiple-domain amnestic MCI patients, who tend to be more severely impaired and likely closer in their transition to AD-type dementia, were excluded. From a predictive modeling standpoint, the exclusion of these MCI cases likely made the task of predicting MCI-to-dementia progression more challenging. To enhance the clinical utility of our predictive model, future work should incorporate data from patients with both amnestic and non-amnestic as well as single-domain and multiple-domain MCI subtypes. Second, our model was specifically designed to predict progression from MCI to AD. In practice, there are multiple other types of dementia in addition to AD (e.g. dementia with Lewy bodies, frontotemporal dementia, vascular dementia), and many cases of dementia are of a mixed etiology (e.g. AD combined with vascular dementia). The probabilistic pattern classification approach adopted in this study can be naturally extended for use in the differential diagnosis of dementia, such that a multi-class classifier could be designed to assign a probability for each type of dementia. Third, we considered only clinical, structural MRI, and plasma proteomic data in this study. Our pattern classification approach could also be applied to biomarker sources such as CSF, PET, and other neuroimaging data. The incorporation of imaging measures of brain connectivity, such as those based on diffusion tensor imaging [86] and resting-state functional MRI [87], may add further predictive information to our model. Furthermore, the incorporation of PET-based amyloid imaging [88] may be particularly useful for improving our model's ability to identify MCI patients who progress to AD more than 18 months after baseline. Finally, we evaluated the predictive performance of our models using cross-validation, a form of internal validation in which a model is developed and evaluated using the same dataset. As the next step, it will be important to externally validate our model on an independent dataset [18,59].

## Conclusions

In summary, we developed a model for predicting progression from MCI to AD-type dementia during a three-year period using a probabilistic, kernel-based pattern classification approach and data from 259 patients with MCI. Using cognitive/functional markers and morphometric MRI markers, the model predicted progression in individual patients with a cross-validated accuracy of 80% and reliably estimated the actual risk of progression. The predictive accuracy of the model varied with demographic, genetic, and clinical characteristics and could be further improved by taking into account the confidence of the predictions. Our prognostic model can potentially improve patient selection in clinical trials and identify high-risk MCI patients for early treatment.

## Supporting Information

S1 Fig Comparison between included (n = 259) and excluded (n = 131) MCI subjects on baseline predictor variables. Error bars are 95% confidence intervals. No group differences were found for any of the predictor variables (all P > 0.4). Vol. = volume, CT = cortical thickness, ADAS-Cog = Alzheimer's Disease Assessment Scale–Cognitive sub-scale, FAQ = Functional Activities Questionnaire, RAVLT = Rey Auditory-Verbal Learning Test, L. = Left, Constr. = Constructional
(PDF)

S1 File Supplemental Materials and Methods. (PDF)

S1 Table Cross-validated performance estimates for single-kernel (5) and multiple-kernel (6–9) multi-source models. In terms of classification accuracy (T-BAR), the CONCAT model performed similarly to MKL-LPG, MKL-Poly, and MKL-Gaussian models (all P > 0.3, paired-sample t-test) and outperformed the MKL-Linear model (P < 0.001). While MKL-LPG and MKL-Poly models were as equally well-calibrated as the CONCAT model (as indicated by the CCC; both P > 0.2), the MKL-Linear model was less well calibrated (P < 0.01) and the MKL-Gaussian model was better calibrated (P < 0.05) than the CONCAT model.
(PDF)

## Abbreviations

ADAS-Cog: Alzheimer's Disease Assessment Scale–Cognitive sub-scale
ADNI: Alzheimer's Disease Neuroimaging Initiative
APOE: apolipoprotein E
AUC: area under the receiver operating characteristic curve
CCC: concordance correlation coefficient
CDR: Clinical Dementia Rating scale
CSF: cerebrospinal fluid
FAQ: Functional Activities Questionnaire
MCI: mild cognitive impairment
MKL: multiple kernel learning
MMSE: Mini-Mental State Examination
MRI: magnetic resonance imaging
NINCDS-ADRDA: National Institute of Neurological and Communicative Disorders and Stroke—Alzheimer's Disease and Related Disorders Association
N-MCI: non-progressive MCI
P-MCI: progressive MCI
PET: positron emission tomography
RAVLT: Rey Auditory Verbal Learning Test
ROI: region of interest

## References

[1] Barnes DE, Yaffe K. The projected effect of risk factor reduction on Alzheimer’s disease prevalence. Lancet Neurol. 2011;10: 819–828. 10.1016/S1474-4422(11)70072-2 21775213 PMC3647614

[2] Holtzman DM, Morris JC, Goate AM. Alzheimer’s disease: the challenge of the second century. Sci Transl Med. 2011;3: 77sr1 10.1126/scitranslmed.3002369 21471435 PMC3130546

[3] Petersen RC, Roberts RO, Knopman DS, Boeve BF, Geda YE, Ivnik RJ, et al Mild cognitive impairment: ten years later. Arch Neurol. 2009;66: 1447–1455. 10.1001/archneurol.2009.266 20008648 PMC3081688

[4] Korolev IO. Alzheimer’s Disease: A Clinical and Basic Science Review. Medical Student Research Journal. 2014;4: 24–33.

[5] Manly JJ, Tang M-X, Schupf N, Stern Y, Vonsattel J-PG, Mayeux R. Frequency and course of mild cognitive impairment in a multiethnic community. Ann Neurol. 2008;63: 494–506. 10.1002/ana.21326 18300306 PMC2375143

[6] Mitchell AJ, Shiri-Feshki M. Rate of progression of mild cognitive impairment to dementia—meta-analysis of 41 robust inception cohort studies. Acta Psychiatr Scand. 2009;119: 252–265. 10.1111/j.1600-0447.2008.01326.x 19236314

[7] Klöppel S, Stonnington CM, Chu C, Draganski B, Scahill RI, Rohrer JD, et al Automatic classification of MR scans in Alzheimer’s disease. Brain. 2008;131: 681–689. 10.1093/brain/awm319 18202106 PMC2579744

[8] Perrin RJ, Fagan AM, Holtzman DM. Multimodal techniques for diagnosis and prognosis of Alzheimer’s disease. Nature. 2009;461: 916–922. 10.1038/nature08538 19829371 PMC2810658

[9] Chen R, Herskovits EH. Machine-learning techniques for building a diagnostic model for very mild dementia. NeuroImage. 2010;52: 234–244. 10.1016/j.neuroimage.2010.03.084 20382237 PMC2917811

[10] Haller S, Lovblad KO, Giannakopoulos P. Principles of classification analyses in mild cognitive impairment (MCI) and Alzheimer disease. J Alzheimers Dis. 2011;26 Suppl 3: 389–394. 10.3233/JAD-2011-0014 21971478

[11] Klöppel S, Abdulkadir A, Jack CR Jr, Koutsouleris N, Mourão-Miranda J, Vemuri P. Diagnostic neuroimaging across diseases. NeuroImage. 2012;61: 457–463. 10.1016/j.neuroimage.2011.11.002 22094642 PMC3420067

[12] Flicker C, Ferris SH, Reisberg B. Mild cognitive impairment in the elderly: predictors of dementia. Neurology. 1991;41: 1006–1009. 2067629 10.1212/wnl.41.7.1006

[13] Tierney MC, Szalai JP, Snow WG, Fisher RH, Nores A, Nadon G, et al Prediction of probable Alzheimer’s disease in memory-impaired patients: A prospective longitudinal study. Neurology. 1996;46: 661–665. 8618663 10.1212/wnl.46.3.661

[14] Kluger A, Ferris SH, Golomb J, Mittelman MS, Reisberg B. Neuropsychological prediction of decline to dementia in nondemented elderly. J Geriatr Psychiatry Neurol. 1999;12: 168–179. 10616864 10.1177/089198879901200402

[15] De Leon MJ, George AE, Stylopoulos LA, Smith G, Miller DC. Early marker for Alzheimer’s disease: the atrophic hippocampus. Lancet. 1989;2: 672–673. 10.1016/s0140-6736(89)90911-2 2570916

[16] De Leon MJ, Golomb J, George AE, Convit A, Tarshish CY, McRae T, et al The radiologic prediction of Alzheimer disease: the atrophic hippocampal formation. AJNR Am J Neuroradiol. 1993;14: 897–906. 8352162 PMC8333817

[17] Jack CR, Petersen RC, Xu YC, O’Brien PC, Smith GE, Ivnik RJ, et al Prediction of AD with MRI-based hippocampal volume in mild cognitive impairment. Neurology. 1999;52: 1397–1403. 10227624 10.1212/wnl.52.7.1397 PMC2730146

[18] Steyerberg EW, Moons KGM, van der Windt DA, Hayden JA, Perel P, Schroter S, et al Prognosis Research Strategy (PROGRESS) 3: Prognostic Model Research. PLoS Med. 2013;10: e1001381 10.1371/journal.pmed.1001381 23393430 PMC3564751

[19] Weiner MW, Veitch DP, Aisen PS, Beckett LA, Cairns NJ, Green RC, et al The Alzheimer’s Disease Neuroimaging Initiative: a review of papers published since its inception. Alzheimers Dement. 2012;8: S1–68. 10.1016/j.jalz.2011.09.172 22047634 PMC3329969

[20] Ben-Hur A, Ong CS, Sonnenburg S, Schölkopf B, Rätsch G. Support vector machines and kernels for computational biology. PLoS Comput Biol. 2008;4: e1000173 10.1371/journal.pcbi.1000173 18974822 PMC2547983

[21] Hofmann T, Schölkopf B, Smola AJ. Kernel methods in machine learning. Ann Statist. 2008;36: 1171–1220. 10.1214/009053607000000677

[22] Gönen M, Alpaydın E. Multiple Kernel Learning Algorithms. J Mach Learn Res. 2011;12: 2211–2268.

[23] Hinrichs C, Singh V, Xu G, Johnson SC. Predictive Markers for AD in a Multi-Modality Framework: An Analysis of MCI Progression in the ADNI Population. Neuroimage. 2011;55: 574–589. 10.1016/j.neuroimage.2010.10.081 21146621 PMC3035743

[24] Zhang D, Shen D. Multi-Modal Multi-Task Learning for Joint Prediction of Multiple Regression and Classification Variables in Alzheimer’s Disease. Neuroimage. 2012;59: 895–907. 10.1016/j.neuroimage.2011.09.069 21992749 PMC3230721

[25] Zhang D, Shen D. Predicting future clinical changes of MCI patients using longitudinal and multimodal biomarkers. PLoS ONE. 2012;7: e33182 10.1371/journal.pone.0033182 22457741 PMC3310854

[26] Cui Y, Liu B, Luo S, Zhen X, Fan M, Liu T, et al Identification of Conversion from Mild Cognitive Impairment to Alzheimer’s Disease Using Multivariate Predictors. PLoS One. 2011;6: e21896 10.1371/journal.pone.0021896 21814561 PMC3140993

[27] Davatzikos C, Bhatt P, Shaw LM, Batmanghelich KN, Trojanowski JQ. Prediction of MCI to AD conversion, via MRI, CSF biomarkers, pattern classification. Neurobiol Aging. 2011;32: 2322.e19–2322.e27. 10.1016/j.neurobiolaging.2010.05.023 PMC2951483 20594615

[28] Westman E, Muehlboeck J-S, Simmons A. Combining MRI and CSF measures for classification of Alzheimer’s disease and prediction of mild cognitive impairment conversion. Neuroimage. 2012;62: 229–238. 10.1016/j.neuroimage.2012.04.056 22580170

[29] Westover MB, Westover KD, Bianchi MT. Significance testing as perverse probabilistic reasoning. BMC Med. 2011;9: 20 10.1186/1741-7015-9-20 21356064 PMC3058025

[30] Damoulas T, Girolami MA. Probabilistic multi-class multi-kernel learning: on protein fold recognition and remote homology detection. Bioinformatics. 2008;24: 1264–1270. 10.1093/bioinformatics/btn112 18378524

[31] Herbei R, Wegkamp MH. Classification with reject option. Canadian Journal of Statistics. 2006;34: 709–721. 10.1002/cjs.5550340410

[32] Petersen RC. Mild cognitive impairment as a diagnostic entity. J Intern Med. 2004;256: 183–194. 10.1111/j.1365-2796.2004.01388.x 15324362

[33] McKhann G, Drachman D, Folstein M, Katzman R, Price D, Stadlan EM. Clinical diagnosis of Alzheimer’s disease: report of the NINCDS-ADRDA Work Group under the auspices of Department of Health and Human Services Task Force on Alzheimer’s Disease. Neurology. 1984;34: 939–944. 6610841 10.1212/wnl.34.7.939

[34] Schneider LS, Insel PS, Weiner MW. Treatment with cholinesterase inhibitors and memantine of patients in the Alzheimer’s Disease Neuroimaging Initiative. Arch Neurol. 2011;68: 58–66. 10.1001/archneurol.2010.343 21220675 PMC3259850

[35] Gomar JJ, Bobes-Bascaran MT, Conejero-Goldberg C, Davies P, Goldberg TE. Utility of Combinations of Biomarkers, Cognitive Markers, and Risk Factors to Predict Conversion From Mild Cognitive Impairment to Alzheimer Disease in Patients in the Alzheimer’s Disease Neuroimaging Initiative. Arch Gen Psychiatry. 2011;68: 961–969. 10.1001/archgenpsychiatry.2011.96 21893661

[36] Palmqvist S, Hertze J, Minthon L, Wattmo C, Zetterberg H, Blennow K, et al Comparison of Brief Cognitive Tests and CSF Biomarkers in Predicting Alzheimer’s Disease in Mild Cognitive Impairment: Six-Year Follow-Up Study. PLoS ONE. 2012;7: e38639 10.1371/journal.pone.0038639 22761691 PMC3382225

[37] Gomar JJ, Conejero-Goldberg C, Davies P, Goldberg TE, Alzheimer’s Disease Neuroimaging Initiative. Extension and refinement of the predictive value of different classes of markers in ADNI: Four-year follow-up data. Alzheimer’s & Dementia: The Journal of the Alzheimer’s Association. 2014; 10.1016/j.jalz.2013.11.009 PMC4416649 24613706

[38] Karow DS, McEvoy LK, Fennema-Notestine C, Hagler DJ, Jennings RG, Brewer JB, et al Relative capability of MR imaging and FDG PET to depict changes associated with prodromal and early Alzheimer disease. Radiology. 2010;256: 932–942. 10.1148/radiol.10091402 20720076 PMC2923729

[39] Jack CR, Bernstein MA, Fox NC, Thompson P, Alexander G, Harvey D, et al The Alzheimer’s Disease Neuroimaging Initiative (ADNI): MRI methods. J Magn Reson Imaging. 2008;27: 685–691. 10.1002/jmri.21049 18302232 PMC2544629

[40] Dale AM, Fischl B, Sereno MI. Cortical surface-based analysis. I. Segmentation and surface reconstruction. Neuroimage. 1999;9: 179–194. 10.1006/nimg.1998.0395 9931268

[41] Desikan RS, Ségonne F, Fischl B, Quinn BT, Dickerson BC, Blacker D, et al An automated labeling system for subdividing the human cerebral cortex on MRI scans into gyral based regions of interest. Neuroimage. 2006;31: 968–980. 10.1016/j.neuroimage.2006.01.021 16530430

[42] Fischl B, Sereno MI, Dale AM. Cortical surface-based analysis. II: Inflation, flattening, and a surface-based coordinate system. Neuroimage. 1999;9: 195–207. 10.1006/nimg.1998.0396 9931269

[43] Fischl B, Salat DH, Busa E, Albert M, Dieterich M, Haselgrove C, et al Whole brain segmentation: automated labeling of neuroanatomical structures in the human brain. Neuron. 2002;33: 341–355. 11832223 10.1016/s0896-6273(02)00569-x

[44] Desikan RS, Cabral HJ, Hess CP, Dillon WP, Glastonbury CM, Weiner MW, et al Automated MRI measures identify individuals with mild cognitive impairment and Alzheimer’s disease. Brain. 2009;132: 2048–2057. 10.1093/brain/awp123 19460794 PMC2714061

[45] Salat DH, Greve DN, Pacheco JL, Quinn BT, Helmer KG, Buckner RL, et al Regional white matter volume differences in nondemented aging and Alzheimer’s disease. Neuroimage. 2009;44: 1247–1258. 10.1016/j.neuroimage.2008.10.030 19027860 PMC2810540

[46] Shen L, Saykin AJ, Kim S, Firpi HA, West JD, Risacher SL, et al Comparison of manual and automated determination of hippocampal volumes in MCI and early AD. Brain Imaging Behav. 2010;4: 86–95. 10.1007/s11682-010-9088-x 20454594 PMC2863347

[47] Graff-Radford NR, Crook JE, Lucas J, Boeve BF, Knopman DS, Ivnik RJ, et al Association of low plasma Abeta42/Abeta40 ratios with increased imminent risk for mild cognitive impairment and Alzheimer disease. Arch Neurol. 2007;64: 354–362. 10.1001/archneur.64.3.354 17353377

[48] Ray S, Britschgi M, Herbert C, Takeda-Uchimura Y, Boxer A, Blennow K, et al Classification and prediction of clinical Alzheimer’s diagnosis based on plasma signaling proteins. Nat Med. 2007;13: 1359–1362. 10.1038/nm1653 17934472

[49] Hansson O, Zetterberg H, Vanmechelen E, Vanderstichele H, Andreasson U, Londos E, et al Evaluation of plasma Abeta(40) and Abeta(42) as predictors of conversion to Alzheimer’s disease in patients with mild cognitive impairment. Neurobiol Aging. 2010;31: 357–367. 10.1016/j.neurobiolaging.2008.03.027 18486992

[50] Johnstone D, Milward EA, Berretta R, Moscato P. Multivariate protein signatures of pre-clinical Alzheimer’s disease in the Alzheimer’s disease neuroimaging initiative (ADNI) plasma proteome dataset. PLoS ONE. 2012;7: e34341 10.1371/journal.pone.0034341 22485168 PMC3317783

[51] Soares HD, Potter WZ, Pickering E, Kuhn M, Immermann FW, Shera DM, et al Plasma Biomarkers Associated With the Apolipoprotein E Genotype and Alzheimer Disease. Archives of Neurology. 2012; 1–8. 10.1001/archneurol.2012.1070 PMC3683865 22801723

[52] Iwatsubo T, Odaka A, Suzuki N, Mizusawa H, Nukina N, Ihara Y. Visualization of A beta 42(43) and A beta 40 in senile plaques with end-specific A beta monoclonals: evidence that an initially deposited species is A beta 42(43). Neuron. 1994;13: 45–53. 8043280 10.1016/0896-6273(94)90458-8

[53] Saeys Y, Inza I, Larrañaga P. A review of feature selection techniques in bioinformatics. Bioinformatics. 2007;23: 2507–2517. 10.1093/bioinformatics/btm344 17720704

[54] Yang HH, Moody J. Feature Selection Based on Joint Mutual Information. Proceedings of International ICSC Symposium on Advances in Intelligent Data Analysis. 1999. pp. 22–25.

[55] Brown G, Pocock A, Zhao M-J, Luján M. Conditional Likelihood Maximisation: A Unifying Framework for Information Theoretic Feature Selection. J Mach Learn Res. 2012;13: 27–66.

[56] Damoulas T, Girolami MA. Combining feature spaces for classification. Pattern Recognition. 2009;42: 2671–2683. 10.1016/j.patcog.2009.04.002

[57] Damoulas T, Girolami MA. Pattern recognition with a Bayesian kernel combination machine. Pattern Recognition Letters. 2009;30: 46–54. 10.1016/j.patrec.2008.08.016

[58] Altman DG, Bland JM. Diagnostic tests. 1: Sensitivity and specificity. BMJ. 1994;308: 1552 8019315 10.1136/bmj.308.6943.1552 PMC2540489

[59] Bouwmeester W, Zuithoff NPA, Mallett S, Geerlings MI, Vergouwe Y, Steyerberg EW, et al Reporting and methods in clinical prediction research: a systematic review. PLoS Med. 2012;9: 1–12. 10.1371/journal.pmed.1001221 PMC3358324 22629234

[60] Kim KI, Simon R. Probabilistic classifiers with high-dimensional data. Biostatistics. 2011;12: 399–412. 10.1093/biostatistics/kxq069 21087946 PMC3138069

[61] Lin LI. A concordance correlation coefficient to evaluate reproducibility. Biometrics. 1989;45: 255–268. 2720055

[62] Altman DG, Bland JM. Diagnostic tests 3: receiver operating characteristic plots. BMJ. 1994;309: 188 8044101 10.1136/bmj.309.6948.188 PMC2540706

[63] Cawley GC, Talbot NLC. On Over-fitting in Model Selection and Subsequent Selection Bias in Performance Evaluation. Journal of Machine Learning Research. 2010;11: 2079–2107.

[64] Smialowski P, Frishman D, Kramer S. Pitfalls of supervised feature selection. Bioinformatics. 2010;26: 440–443. 10.1093/bioinformatics/btp621 19880370 PMC2815655

[65] Varma S, Simon R. Bias in error estimation when using cross-validation for model selection. BMC Bioinformatics. 2006;7: 91 10.1186/1471-2105-7-91 16504092 PMC1397873

[66] Bouckaert RR. Choosing between Two Learning Algorithms Based on Calibrated Tests In ICML’03. Morgan Kaufmann; 2003 pp. 51–58.

[67] Jack CR, Knopman DS, Jagust WJ, Shaw LM, Aisen PS, Weiner MW, et al Hypothetical model of dynamic biomarkers of the Alzheimer’s pathological cascade. Lancet Neurol. 2010;9: 119–128. 10.1016/S1474-4422(09)70299-6 20083042 PMC2819840

[68] Bozoki A, Giordani B, Heidebrink JL, Foster NL. Mild cognitive impairments predict dementia in nondemented elderly patients with memory loss. Arch Neurol. 2001;58: 411–416. 11255444 10.1001/archneur.58.3.411

[69] Bäckman L, Jones S, Berger A-K, Laukka EJ, Small BJ. Cognitive impairment in preclinical Alzheimer’s disease: a meta-analysis. Neuropsychology. 2005;19: 520–531. 10.1037/0894-4105.19.4.520 16060827

[70] Albert MS, DeKosky ST, Dickson D, Dubois B, Feldman HH, Fox NC, et al The diagnosis of mild cognitive impairment due to Alzheimer’s disease: recommendations from the National Institute on Aging-Alzheimer’s Association workgroups on diagnostic guidelines for Alzheimer’s disease. Alzheimers Dement. 2011;7: 270–279. 10.1016/j.jalz.2011.03.008 21514249 PMC3312027

[71] Thompson PM, Hayashi KM, de Zubicaray G, Janke AL, Rose SE, Semple J, et al Dynamics of gray matter loss in Alzheimer’s disease. J Neurosci. 2003;23: 994–1005. 12574429 10.1523/JNEUROSCI.23-03-00994.2003 PMC6741905

[72] Ye J, Farnum M, Yang E, Verbeeck R, Lobanov V, Raghavan N, et al Sparse learning and stability selection for predicting MCI to AD conversion using baseline ADNI data. BMC Neurology. 2012;12: 46 10.1186/1471-2377-12-46 22731740 PMC3477025

[73] Devanand DP, Liu X, Brown PJ, Huey ED, Stern Y, Pelton GH. A two-study comparison of clinical and MRI markers of transition from mild cognitive impairment to Alzheimer’s disease. Int J Alzheimers Dis. 2012;2012: 483469 10.1155/2012/483469 22482070 PMC3296186

[74] Devanand DP, Liu X, Tabert MH, Pradhaban G, Cuasay K, Bell K, et al Combining early markers strongly predicts conversion from mild cognitive impairment to Alzheimer’s disease. Biol Psychiatry. 2008;64: 871–879. 10.1016/j.biopsych.2008.06.020 18723162 PMC2613777

[75] Young J, Modat M, Cardoso MJ, Mendelson A, Cash D, Ourselin S. Accurate multimodal probabilistic prediction of conversion to Alzheimer’s disease in patients with mild cognitive impairment. Neuroimage (Amst). 2013;2: 735–745. 10.1016/j.nicl.2013.05.004 PMC3777690 24179825

[76] Wee C-Y, Yap P-T, Shen D, Alzheimer’s Disease Neuroimaging Initiative. Prediction of Alzheimer’s disease and mild cognitive impairment using cortical morphological patterns. Hum Brain Mapp. 2013;34: 3411–3425. 22927119 10.1002/hbm.22156 PMC3511623

[77] Raji CA, Lopez OL, Kuller LH, Carmichael OT, Becker JT. Age, Alzheimer disease, and brain structure. Neurology. 2009;73: 1899–1905. 10.1212/WNL.0b013e3181c3f293 19846828 PMC2788799

[78] Stricker NH, Chang Y-L, Fennema-Notestine C, Delano-Wood L, Salmon DP, Bondi MW, et al Distinct profiles of brain and cognitive changes in the very old with Alzheimer disease. Neurology. 2011;77: 713–721. 10.1212/WNL.0b013e31822b0004 21832223 PMC3164395

[79] Wishart HA, Saykin AJ, McAllister TW, Rabin LA, McDonald BC, Flashman LA, et al Regional brain atrophy in cognitively intact adults with a single APOE ε4 allele. Neurology. 2006;67: 1221–1224. 10.1212/01.wnl.0000238079.00472.3a 17030756

[80] Koikkalainen J, Pölönen H, Mattila J, van Gils M, Soininen H, Lötjönen J, et al Improved Classification of Alzheimer’s Disease Data via Removal of Nuisance Variability. PLoS ONE. 2012;7: e31112 10.1371/journal.pone.0031112 22348041 PMC3278425

[81] Sperling RA, Rentz DM, Johnson KA, Karlawish J, Donohue M, Salmon DP, et al The A4 study: stopping AD before symptoms begin? Sci Transl Med. 2014;6: 228fs13 10.1126/scitranslmed.3007941 24648338 PMC4049292

[82] Beach TG, Monsell SE, Phillips LE, Kukull W. Accuracy of the clinical diagnosis of Alzheimer disease at National Institute on Aging Alzheimer Disease Centers, 2005–2010. J Neuropathol Exp Neurol. 2012;71: 266–273. 10.1097/NEN.0b013e31824b211b 22437338 PMC3331862

[83] Salloway S, Sperling R, Fox NC, Blennow K, Klunk W, Raskind M, et al Two phase 3 trials of bapineuzumab in mild-to-moderate Alzheimer’s disease. N Engl J Med. 2014;370: 322–333. 10.1056/NEJMoa1304839 24450891 PMC4159618

[84] Mitchell AJ, Shiri-Feshki M. Temporal trends in the long term risk of progression of mild cognitive impairment: a pooled analysis. J Neurol Neurosurg Psychiatr. 2008;79: 1386–1391. 10.1136/jnnp.2007.142679 19010949

[85] Lambert JC, Ibrahim-Verbaas CA, Harold D, Naj AC, Sims R, Bellenguez C, et al Meta-analysis of 74,046 individuals identifies 11 new susceptibility loci for Alzheimer’s disease. Nat Genet. 2013;45: 1452–1458. 10.1038/ng.2802 24162737 PMC3896259

[86] Bozoki AC, Korolev IO, Davis NC, Hoisington LA, Berger KL. Disruption of limbic white matter pathways in mild cognitive impairment and Alzheimer’s disease: a DTI/FDG-PET study. Hum Brain Mapp. 2012;33: 1792–1802. 10.1002/hbm.21320 21674695 PMC6870438

[87] Zhu DC, Majumdar S, Korolev IO, Berger KL, Bozoki AC. Alzheimer’s disease and amnestic mild cognitive impairment weaken connections within the default-mode network: a multi-modal imaging study. J Alzheimers Dis. 2013;34: 969–984. 10.3233/JAD-121879 23313926

[88] Nordberg A, Carter SF, Rinne J, Drzezga A, Brooks DJ, Vandenberghe R, et al A European multicentre PET study of fibrillar amyloid in Alzheimer’s disease. Eur J Nucl Med Mol Imaging. 2013;40: 104–114. 10.1007/s00259-012-2237-2 22961445 PMC3510420

