# Supplementary material for: Predicting Progression from Mild Cognitive Impairment to Alzheimer's Dementia Using Clinical, MRI, and Plasma Biomarkers via Probabilistic Pattern Classification
Source: PLoS One. 2016 Feb 22;11(2):e0138866. doi: 10.1371/journal.pone.0138866 (PMC4762666; doi:10.1371/journal.pone.0138866)
Supplement: S1 Fig — Error bars are 95% confidence intervals. No group differences were found for any of the predictor variables (all P > 0.4). Vol. = volume, CT = cortical thickness, ADAS-Cog = Alzheimer's Disease Assessment Scale–Cognitive sub-scale, FAQ = Functional Activities Questionnaire, RAVLT = Rey Auditory-Verbal Learning Test, L. = Left, Constr. = Constructional (PDF) [file pone.0138866.s001.pdf]

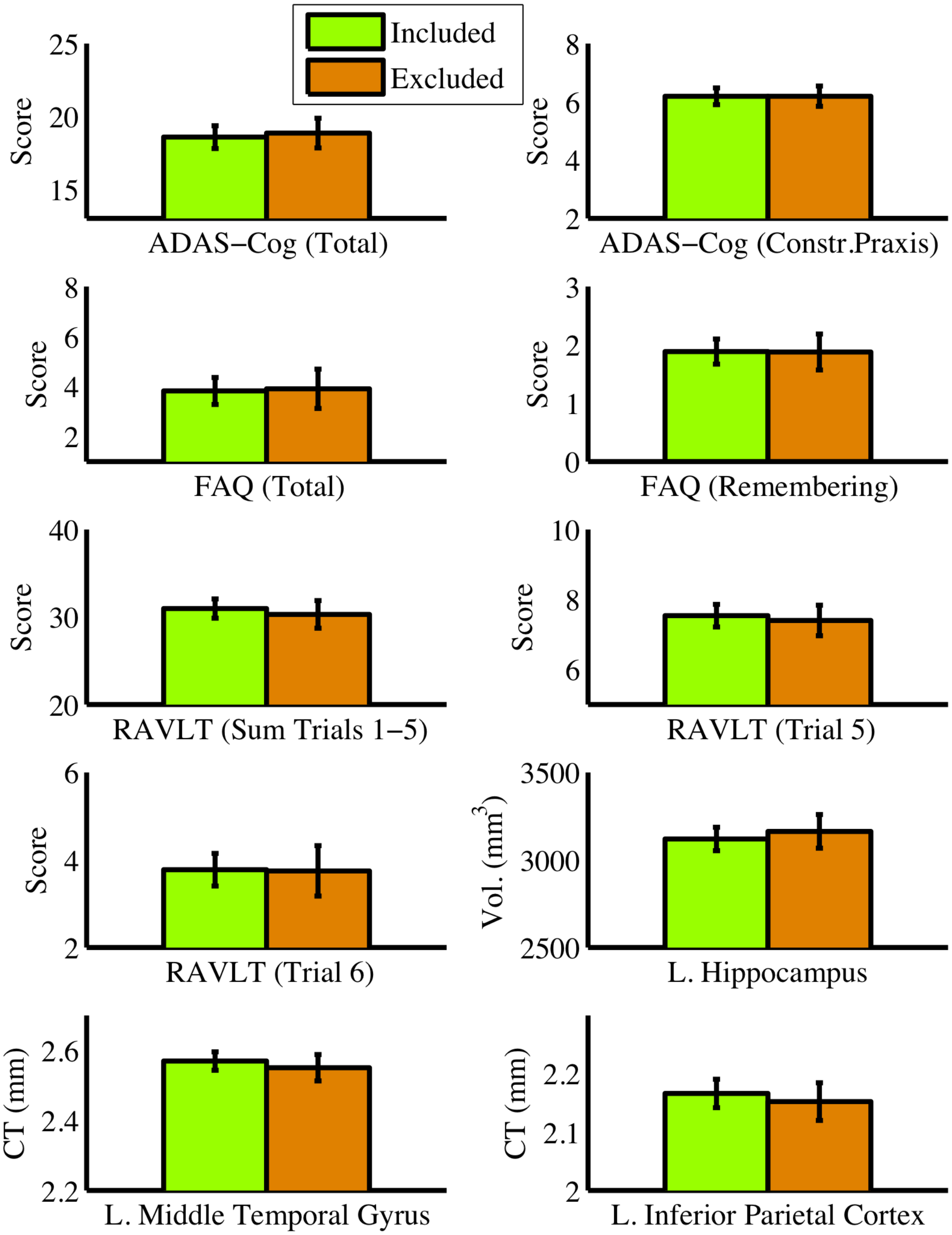

Figure S1. Comparison between included (n=259) and excluded (n=131) MCI subjects on baseline predictor variables. Error bars are 95% confidence intervals. No group differences were found for any of the predictor variables (all $P > 0.4$). Vol. = volume, CT = cortical thickness, ADAS-Cog = Alzheimer's Disease Assessment Scale – Cognitive sub-scale, FAQ = Functional Activities Questionnaire, RAVLT = Rey Auditory-Verbal Learning Test, L. = Left, Constr. = Constructional
