# Supplementary material for: Predicting Progression from Mild Cognitive Impairment to Alzheimer's Dementia Using Clinical, MRI, and Plasma Biomarkers via Probabilistic Pattern Classification
Source: PLoS One. 2016 Feb 22;11(2):e0138866. doi: 10.1371/journal.pone.0138866 (PMC4762666; doi:10.1371/journal.pone.0138866)
Supplement: S1 File — (PDF) [file pone.0138866.s002.pdf]

**Supplement 1: Materials and Methods**

## *1.1 General Eligibility Criteria for Subjects in ADNI*

Enrolled subjects were 55 to 90 years old with a minimum 6th-grade level of education; had a study partner able to provide an independent evaluation of functioning; could speak either English or Spanish; had adequate visual and auditory acuity to allow neuropsychological testing; were willing and able to undergo all test procedures, including neuroimaging; and agreed to longitudinal follow-up. All subjects had no significant neurologic disease, major depression, history of schizophrenia or bipolar disorder, recent history of alcohol or substance abuse, and no pacemakers or other objects deemed unsafe for MRI.

## *1.2 Clinical Data (additional details)*

*Clinical Risk Factors*

1. Age (years)
2. Sex (Male/Female)
3. Education (years)
4. APOE genotype:
    a. Number of ε4 alleles
    b. ε4 allele carrier status
5. Family history of dementia:
    a. Parental
    b. Maternal
    c. Paternal
6. Number of cerebrovascular disease risk factors (0-6):
   [1] Diabetes mellitus, [2] cardiovascular disease (coronary/carotid artery disease, valvular disease, congestive heart failure), [3] hypertension, [4] smoking (>5 pack-years), [5] hyperlipidemia, [6] stroke/TIA
7. Body mass index

8. History of:
    a. Depression
    b. Anxiety
    c. Other psychiatric disorders
    d. Alcohol abuse
    e. Head trauma (with loss of consciousness)
    f. Sleep apnea

*Clinical Assessments*

The following assessments were administered to the subjects in ADNI. The Mini-Mental State Examination (MMSE) (Folstein et al., 1975) is widely used as a screening test for dementia and assesses cognitive function in multiple domains, including orientation, language, attention, calculation, constructional praxis, and memory. The Clinical Dementia Rating (CDR) scale (Morris, 1993) is administered as a semi-structured interview with both the patient and an informant that assesses the patient's functional and cognitive status in six domains: memory, orientation, judgment and problem-solving, community affairs, home and hobbies, and personal care. The Functional Activities Questionnaire (FAQ) (Pfeffer et al., 1982) assesses the level of independence in performing activities of daily living (e.g. record keeping, managing finances, shopping, meal preparation, remembering dates, transportation). The Geriatric Depression Scale (GDS) (Sheikh and Yesavage, 1986) is a self-report assessment of depressive symptoms and designed to be used as a screening test for depression in older adults. The Neuropsychiatric Inventory Questionnaire (NPI-Q) (Kaufer et al., 2000) is an informant-based assessment of recent psychiatric and behavioral symptoms (e.g. hallucinations, agitation, depression, anxiety, apathy, disinhibition, irritability). The Modified Hachinski Ischemic Scale (HIS) (Rosen et al., 1980) assesses the contribution of cerebrovascular disease to cognitive impairment based on medical history and neurological

symptoms and signs. The American National Adult Reading Test (ANART) (Grober et al., 1991) provides a measure of premorbid intelligence by assessing pronunciation of 50 irregular words.

In addition, a battery of neuropsychological tests was administered to further evaluate function in specific cognitive domains. The WMS-III Logical Memory (LM) is a test of episodic memory function that assesses immediate and delayed story recall (Johnson et al., 2003). The Alzheimer's Disease Assessment Scale – Cognitive sub-scale (ADAS-Cog) (Mohs et al., 1997) assesses multiple aspects of memory and language function as well as attention, orientation, and praxis. The Rey Auditory Verbal Learning Test (RAVLT) (Vakil and Blachstein, 1993) involves learning lists of words and assesses verbal memory. The verbal (category) fluency test (Acevedo et al., 2000) and Boston Naming Test (BNT) (Zec et al., 2007) assess semantic memory and language function. The digit span test (Hester et al., 2004) assesses verbal working memory. The Trail Making Test (TMT) (Tombaugh, 2004) evaluates processing speed (part A) and executive function (part B). The Digit-Symbol Coding Test (DST) (Joy et al., 2004) assesses processing speed, visual working memory, and visual-motor coordination. The Clock-Drawing Test (CDT) (Shulman, 2000) assesses constructional praxis with elements of visuospatial and executive function.

### *1.3 MRI Data Acquisition and Processing*

*MRI Acquisition Parameters:* T1-weighted sagittal 3-D MP-RAGE sequence with 1.25 x 1.25 $mm^2$ in-plane resolution and 1.2 mm slice thickness, TR = 2400 ms, TI = 1000 ms, TE = 3 ms, flip angle = 8°, 240 x 240 $mm^2$ FOV, 192 x 192 in-plain matrix size. Raw MRI data underwent quality control and were pre-processed by the ADNI MRI Core to correct for image geometry distortion due to gradient non-linearity and image intensity non-uniformity (see

www.adni-info.org for details). In this study, we used these pre-processed, corrected MRI datasets.

*FreeSurfer Processing Steps:* removal of non-brain tissue (skull stripping); Talairach transformation of the brain volume into standard anatomical space; intensity normalization; segmentation of cerebral white matter (WM), subcortical gray matter (GM), and ventricles; delineation and 3-D reconstruction of GM/WM and GM/CSF (cerebrospinal fluid) boundaries; and automated topology correction. The cortical surface model was registered to a spherical atlas and used for segmentation of the cerebral cortex into regions based on gyral-sulcal anatomy (i.e. cortical folding pattern). The final segmentation and labeling of brain structures was based on a probabilistic atlas along with intensity and curvature information.

### *1.4 Data Transformation*

First, volumetric and surface area MRI measures were normalized by the estimated total intracranial volume (Buckner et al., 2004) to correct for individual differences in head size. Second, each of the 787 features was scaled to have zero mean and unit variance across subjects. Third, each continuous and ordinal feature was discretized into three states (low, intermediate, high) using the mean and standard deviation to define interval boundaries, as described in (Ding and Peng, 2005). Discretized features were used only when conducting information-theoretic feature selection (described below) while non-discretized features were used during model training.

### *1.5 Feature Selection (additional details)*

*Background*: Feature selection is an important component of the model development process, particularly in the case of high-dimensional pattern classification where the number

of features is large and exceeds the number of samples available for classification (787 features and 259 subjects in this study). Many of these features may be irrelevant, redundant, or noisy. Feature selection techniques include filter- and wrapper-based methods. Filter methods tend to be fast and identify informative features based on inherent statistical properties of the data, independent of any classifier. In contrast, wrapper methods evaluate the merit of various feature subsets based on the performance of a classifier and may select features for classification more effectively, although at a significant cost in terms of speed and greater potential for overfitting.

*Joint Mutual Information (JMI)*: JMI is a multivariate information-theoretic filter method for feature selection and has been shown to perform well in terms of both classification accuracy and stability on a wide range of real-world datasets (Brown et al., 2012). Features are selected based on their JMI score (*J*), defined as:

$$J(X_k) = I(X_k;Y) - \frac{1}{|S|}\sum_{j\in S}\left[I(X_k;X_j) - I(X_k;X_j|Y)\right] \quad (1)$$

where $X_k$ is the feature being considered for selection, $X_j$ is each of the previously selected features in feature subset $S$, and $Y$ is the outcome/class variable of interest (future dementia status, in this study). The JMI score for a given feature $X_k$ is defined as a linear combination of three mutual information terms $I$, each of which describes the amount of information shared (or the dependence) between two random variables; these terms correspond to relevance $I(X_k;Y)$, redundancy $I(X_k;X_j)$, and class-conditional redundancy $I(X_k;X_j|Y)$ (Brown et al., 2012).

### *1.6 Pattern Classification Approach (additional details)*

*Kernels:* Different kernel functions can be used to provide varying definitions of similarity. The similarity between a pair of examples $a$ and $b$, described by their feature vectors $x_a$ and $x_b$, can be defined according to each kernel function $K$ as:

$$K(x_a, x_b) = x_a \cdot x_b \quad \text{(linear)} \tag{2}$$

$$K(x_a, x_b) = (x_a \cdot x_b + c)^d \quad \text{(polynomial)} \tag{3}$$

$$K(x_a, x_b) = \exp(-\gamma \|x_a - x_b\|^2) \quad \text{(Gaussian)} \tag{4}$$

where $c$ is a constant, $d$ is the degree of the polynomial, and γ is the kernel width. While an advantage of the linear kernel is that there are no kernel parameters to set, the linear kernel is unable to capture more complex patterns in the data, as can be done by using non-linear kernels. In this study, we build models with both linear and nonlinear (polynomial and Gaussian) kernels.

*Multiple Kernel Learning (MKL):* For illustration, consider a dataset with $N$ examples and $S$ sources or representations of the data, with each example described by the feature vector $x_n^s$ and discrete class (outcome) label $Y_n \in \{1, \ldots, C\}$ where $n = 1, \ldots, N$, $s = 1, \ldots, S$, and $C$ is the number of classes (outcomes). The pMKL classifier integrates this information by learning an optimal linear combination of the multiple kernels (Damoulas and Girolami, 2008), such that the $N \times N$ composite kernel is defined as:

$$K^{\beta\Theta}(\boldsymbol{x_a}, \boldsymbol{x_b}) = \sum_{s=1}^{S} \beta_s K^{s\theta_s}(\boldsymbol{x_a^s}, \boldsymbol{x_b^s}) \tag{5}$$

In Eq. (5), $\beta_s$ is the kernel weight describing the relative contribution of data source (representation) $s$ and $\theta_s$ is the kernel parameter that controls the amount of data smoothing (e.g. degree $d$ of the polynomial kernel or width γ of the Gaussian kernel).

### *1.7 Multiple-kernel Multi-source Models*

In models 6-8, we incorporated different nonlinear kernels in order to capture information regarding more complex interactions among features and to integrate potentially complementary representations of the feature data. These models were constructed by considering all features and data sources simultaneously and included: i) a model with five Gaussian kernels ($\gamma=10^{-2}$, $10^{-1}$, $10^{0}$, $10^{1}$, $10^{2}$) (model 6; 'MKL-Gaussian'); ii) a three-kernel model with a linear, polynomial (d=2 and c=1), and Gaussian ($\gamma=1/D$) kernels, where D is the number of features (model 7; 'MKL-LPG'); and iii) a model with five polynomial kernels (d=1, 2, 3, 4, 5 and c=1) (model 8; 'MKL-Poly'). In model 9 (MKL-Linear), a separate linear kernel was used to encode the most informative features from each of the four data sources, as determined in single-source models 1-4.

### *1.8 Concordance Correlation Coefficient*

The concordance correlation coefficient (CCC) (Lin, 1989) was calculated as a measure of model calibration as follows. The probability interval (0-100%) was divided into 10 equal sub-intervals. Then, the predicted probability of MCI-to-dementia progression (generated by the pMKL classifier and averaged across subjects) and actual probability of progression (fraction of subjects belonging to the P-MCI group) were computed for each of these 10 sub-intervals. The CCC was calculated by comparing these 10 pairs of predicted-actual probability values. The reported CCC value is an average across cross-validation runs. The CCC can range from +1 (perfect agreement) to -1 (perfect disagreement), with values of CCC near zero indicating weak or no relationship between predicted and actual probabilities. By using the 10 probability sub-intervals (minimum recommended in Lin, 1989) we obtained a

robust estimate for the CCC while keeping the sub-intervals sufficiently large to maximize the number of subjects within each sub-interval.

### *1.9 Cross-validation Procedure (additional details)*

*Background:* Cross-validation (CV) (Kohavi, 1995) refers to various data partitioning techniques commonly used in statistics and machine learning fields when developing predictive models and assessing their performance. The key goal of CV is to obtain an unbiased estimate of a model's predictive performance in circumstances of limited data availability. In essence, CV allows one to estimate how well a model can be expected to make predictions in real-world settings on new data.

*Nested stratified 10-fold CV:* In 10-fold stratified CV (Kohavi, 1995), the dataset is randomly partitioned into 10 mutually exclusive parts (folds) of equal size, preserving the proportion of samples in each class as found in the full dataset. Nine out of 10 parts are used to train the classifier, which is then evaluated on the remaining one part. This is repeated until each fold of the dataset has been used once for evaluation, thus resulting in 10 performance estimates per one run of 10-fold CV. During each fold of the outer CV loop, the full dataset (n=259) was split into a 'model development set' (90%) and a 'test set' (10%), which was held out for final model evaluation. Feature selection, model (parameter) selection, and final model construction were repeated independently for each fold of the outer CV loop and based only on the 'model development set'. The inner CV loop was designed to determine the optimal feature subset size for use in the final model. During each fold of the inner CV loop, data from the 'model development set' were split into a 'training set' (90%) and 'validation set' (10%). Then, JMI-based feature selection was performed based only on the 'training set' to identify subsets with the top *D* most informative features for discriminating between N-MCI and P-

MCI, where $D \in \{1, 3, 5, 7, 10, 15, 20, 30, 40, 50\}$. A classifier was then constructed for each of the ten feature subsets using the 'training set' and evaluated on the 'validation set'. The subset size resulting in the highest 10-fold CV accuracy on the 'validation set' was then selected as the optimal feature subset size, $D_{OPTIMAL}$. The final model classifier was constructed using the top $D_{OPTIMAL}$ most informative features (selected via the JMI method) based on the 'model development set' and evaluated on the 'test set'.

**References**

Acevedo, A., Loewenstein, D.A., Barker, W.W., Harwood, D.G., Luis, C., Bravo, M., Hurwitz, D.A., Aguero, H., Greenfield, L., and Duara, R. (2000). Category Fluency Test: Normative data for English- and Spanish-speaking elderly. Journal of the International Neuropsychological Society *6*, 760–769.

Brown, G., Pocock, A., Zhao, M.-J., and Luján, M. (2012). Conditional Likelihood Maximisation: A Unifying Framework for Information Theoretic Feature Selection. J. Mach. Learn. Res. *13*, 27–66.

Buckner, R.L., Head, D., Parker, J., Fotenos, A.F., Marcus, D., Morris, J.C., and Snyder, A.Z. (2004). A unified approach for morphometric and functional data analysis in young, old, and demented adults using automated atlas-based head size normalization: reliability and validation against manual measurement of total intracranial volume. Neuroimage *23*, 724–738.

Damoulas, T., and Girolami, M.A. (2008). Probabilistic multi-class multi-kernel learning: on protein fold recognition and remote homology detection. Bioinformatics *24*, 1264–1270.

Ding, C., and Peng, H. (2005). Minimum redundancy feature selection from microarray gene expression data. J Bioinform Comput Biol *3*, 185–205.

Folstein, M.F., Folstein, S.E., and McHugh, P.R. (1975). “Mini-mental state”. A practical method for grading the cognitive state of patients for the clinician. J Psychiatr Res *12*, 189–198.

Grober, E., Sliwinsk, M., and Korey, S.R. (1991). Development and Validation of a Model for Estimating Premorbid Verbal Intelligence in the Elderly. Journal of Clinical and Experimental Neuropsychology *13*, 933–949.

Hester, R.L., Kinsella, G.J., and Ong, B. (2004). Effect of age on forward and backward span tasks. J Int Neuropsychol Soc *10*, 475–481.

Johnson, D.K., Storandt, M., and Balota, D.A. (2003). Discourse analysis of logical memory recall in normal aging and in dementia of the Alzheimer type. Neuropsychology *17*, 82–92.

Joy, S., Kaplan, E., and Fein, D. (2004). Speed and memory in the WAIS-III Digit Symbol--Coding subtest across the adult lifespan. Arch Clin Neuropsychol *19*, 759–767.

Kaufer, D.I., Cummings, J.L., Ketchel, P., Smith, V., MacMillan, A., Shelley, T., Lopez, O.L., and DeKosky, S.T. (2000). Validation of the NPI-Q, a brief clinical form of the Neuropsychiatric Inventory. J Neuropsychiatry Clin Neurosci *12*, 233–239.

Kohavi, R. (1995). A study of cross-validation and bootstrap for accuracy estimation and model selection. In Proceedings of the 14th International Joint Conference on Artificial Intelligence - Volume 2, (San Francisco, CA, USA: Morgan Kaufmann Publishers Inc.), pp. 1137–1143.

Lin, L.I. (1989). A concordance correlation coefficient to evaluate reproducibility. Biometrics *45*, 255–268.

Mohs, R.C., Knopman, D., Petersen, R.C., Ferris, S.H., Ernesto, C., Grundman, M., Sano, M., Bieliauskas, L., Geldmacher, D., Clark, C., et al. (1997). Development of cognitive instruments for use in clinical trials of antidementia drugs: additions to the Alzheimer's Disease Assessment Scale that broaden its scope. The Alzheimer's Disease Cooperative Study. Alzheimer Dis Assoc Disord *11 Suppl 2*, S13–21.

Morris, J.C. (1993). The Clinical Dementia Rating (CDR): current version and scoring rules. Neurology *43*, 2412–2414.

Pfeffer, R.I., Kurosaki, T.T., Harrah, C.H., Chance, J.M., and Filos, S. (1982). Measurement of Functional Activities in Older Adults in the Community. J Gerontol *37*, 323–329.

Rosen, W.G., Terry, R.D., Fuld, P.A., Katzman, R., and Peck, A. (1980). Pathological verification of ischemic score in differentiation of dementias. Ann. Neurol. *7*, 486–488.

Sheikh, J.I., and Yesavage, J.A. (1986). Geriatric Depression Scale (GDS): recent evidence and development of a shorter version. In Clinical Gerontology : A Guide to Assessment and Intervention, (NY: The Haworth Press), pp. 165–173.

Shulman, K.I. (2000). Clock-drawing: is it the ideal cognitive screening test? Int J Geriatr Psychiatry *15*, 548–561.

Tombaugh, T.N. (2004). Trail Making Test A and B: normative data stratified by age and education. Arch Clin Neuropsychol *19*, 203–214.

Vakil, E., and Blachstein, H. (1993). Rey auditory-verbal learning test: Structure analysis. Journal of Clinical Psychology *49*, 883–890.

Zec, R.F., Burkett, N.R., Markwell, S.J., and Larsen, D.L. (2007). Normative data stratified for age, education, and gender on the Boston Naming Test. Clin Neuropsychol *21*, 617–637.
