# Supplementary material for: Predicting Progression from Mild Cognitive Impairment to Alzheimer's Dementia Using Clinical, MRI, and Plasma Biomarkers via Probabilistic Pattern Classification
Source: PLoS One. 2016 Feb 22;11(2):e0138866. doi: 10.1371/journal.pone.0138866 (PMC4762666; doi:10.1371/journal.pone.0138866)
Supplement: S1 Table — In terms of classification accuracy (T-BAR), the CONCAT model performed similarly to MKL-LPG, MKL-Poly, and MKL-Gaussian models (all P > 0.3, paired-sample t-test) and outperformed the MKL-Linear model (P < 0.001). While MKL-LPG and MKL-Poly models were as equally well-calibrated as the CONCAT model (as indicated by the CCC; both P > 0.2), the MKL-Linear model was less well calibrated (P < 0.01) and the MKL-Gaussian model was better calibrated (P < 0.05) than the CONCAT model. (PDF) [file pone.0138866.s003.pdf]

Model (#)	V-BAR (%)	T-BAR (%)	Sn (%)	Sp (%)	AUC	CCC	$D_{OPTIMAL}$ / Total
CONCAT (5)	79.7 ± 1.4	80.0 ± 7.3	80.3 ± 10.6	79.8 ± 10.9	0.86 ± 0.07	0.93 ± 0.02	10 ± 3 / 787
MKL-Gaussian (6)	80.3 ± 1.3	79.9 ± 6.8	83.4 ± 9.9	76.4 ± 12.3	0.87 ± 0.07	0.95 ± 0.01	10 ± 3 / 787
MKL-LPG (7)	80.2 ± 1.5	79.7 ± 7.2	81.0 ± 9.8	78.3 ± 12.3	0.87 ± 0.07	0.94 ± 0.02	10 ± 0 / 787
MKL-Poly (8)	80.1 ± 1.4	79.5 ± 7.5	82.2 ± 10.2	76.8 ± 12.4	0.87 ± 0.07	0.94 ± 0.02	10 ± 3 / 787
MKL-Linear (9)	##	74.9 ± 6.7	74.6 ± 11.7	75.2 ± 11.9	0.84 ± 0.07	0.88 ± 0.04	74 ± 18 / 787

V-BAR and T-BAR = Validation Set and Test Set Balanced Accuracy Rate, Sn = Sensitivity, Sp = Specificity, AUC = Area under the Curve from Receiver Operating Characteristic analysis, CCC = Concordance Correlation Coefficient, $D_{OPTIMAL}$ = optimal number of features

Model 5 (CONCAT): single linear kernel, concatenating features from all data sources.

Model 6 (MKL-Gaussian): 5 Gaussian kernels using features from all data sources.

Model 7 (MKL-LPG): 3 kernels (linear, polynomial, Gaussian) using features from all data sources.

Model 8 (MKL-Poly): 5 polynomial kernels using features from all data sources.

Model 9 (MKL-Linear): 4 linear kernels, each encoding the most informative features for one of the four data sources (CRF, CAM, MRI, PPM), as determined in single-source models 1-4. ## Since no inner CV was necessary other than what was done in models 1-4 to determine the optimal feature subset size, V-BAR was not calculated for this model.

Table S1. Cross-validated performance estimates for single-kernel (5) and multiple-kernel (6-9) multi-source models. In terms of classification accuracy (T-BAR), the CONCAT model performed similarly to MKL-LPG, MKL-Poly, and MKL-Gaussian models (all $P > 0.3$, paired-sample *t*-test) and outperformed the MKL-Linear model ($P < 0.001$). While MKL-LPG and MKL-Poly models were as equally well-calibrated as the CONCAT model (as indicated by the CCC; both $P > 0.2$), the MKL-Linear model was less well calibrated ($P < 0.01$) and the MKL-Gaussian model was better calibrated ($P < 0.05$) than the CONCAT model.
